# Lightweight and interpretable edge intelligence AI with intrusion detection for trustworthy cardiac arrhythmia in medical IoT

**DOI:** 10.1038/s41598-026-43578-6

**Published:** 2026-03-24

**Authors:** Muhammad Imran Khalid, Altaf Hussain, Nasir Hussain, Tamim Alkhalifah

**Affiliations:** 1 School of Computer Science and Technology, Chongqing University of Posts and Telecommunications, Chongqing, 400065 China; 2 Department of Computer Engineering, College of Computer, Qassim University, Buraydah, Saudi Arabia

**Keywords:** Medical internet of things, Intrusion detection system, Explainable artificial intelligence (XAI), Arrhythmia detection, Interpretable machine learning, CLARITY-AI, Cardiology, Computational biology and bioinformatics, Engineering, Health care, Mathematics and computing

## Abstract

Medical Internet of Things (MIoT) systems enable continuous cardiac monitoring, but practical deployment is limited by three issues: heavy computation at the edge, limited interpretability, and vulnerability to cyber-attacks that can corrupt signals and degrade inference. We propose CLARITY-AI 2.0, a lightweight and trustworthy arrhythmia detection framework for MIoT that combines efficient ECG feature extraction, interpretable prediction, and security-aware trust assessment. The model performs arrhythmia inference using a compact feature-based learner and generates clinician-facing explanations via SHAP attributions, which are converted into structured natural-language reports using an LLM. To improve trust under hostile network conditions, an intrusion detection module outputs an attack probability and a trust score that flags unreliable inputs. On the MIT-BIH benchmark, CLARITY-AI 2.0 achieves an F1 score of 0.928 and an AUC of 0.985 for anomaly detection. It also generalizes under dataset shift with an AUC of 0.940 on PTB-XL and an AUC of 0.915 on Chapman (zero-shot evaluation). For edge feasibility, deployment on an ESP32 reduces the footprint to 350 KB, peak RAM to 120 KB, and inference latency to 8.1 ms, supporting real-time operation on resource-constrained devices. These results indicate that CLARITY-AI 2.0 is an interpretable, efficient, and security-aware approach toward scalable MIoT-based cardiac monitoring.

## Introduction

Cardiovascular diseases (CVDs) remain the leading cause of mortality worldwide, with cardiac arrhythmias serving as a primary indicator of adverse and potentially fatal cardiac events^1^. Timely detection of arrhythmic episodes is therefore critical, as delays in diagnosis, particularly in emergency scenarios, can lead to severe complications and increased mortality rates^2^. Conventional arrhythmia assessment using 12-lead Holter monitoring is resource-intensive, episodic, and limited in duration, restricting its ability to support continuous and preventive cardiac care. In contrast, recent advances in MIoT and wearable sensing technologies have enabled continuous, real-time electrocardiogram (ECG) monitoring, facilitating early disease detection and personalized health management^3,4^. The integration of wearable devices into cardiovascular medicine is no longer conceptual but has emerged as a rapidly growing and clinically impactful paradigm^5^. This technological shift has catalyzed extensive research in automated ECG analysis using advanced artificial intelligence (AI) techniques. Recent studies demonstrate high arrhythmia classification performance using Deep Learning (DL) architectures, including 1D Convolutional Neural Networks (CNNs), attention-based models such as CardioAttentionNet^6^, Transformer-based hybrid architectures^3,7^, and even object-detection paradigms such as YOLOv8^8^. Beyond signal-level analysis, multimodal AI approaches have further leveraged patient metadata to predict arrhythmic mortality risk^9^. Despite their impressive accuracy, these increasingly complex DL models face substantial barriers to real-world deployment in clinical and edge-based MIoT environments.

The first major limitation is the lack of interpretability inherent in deep learning models. While highly accurate, most DL approaches operate as black boxes, producing diagnostic labels (e.g., “arrhythmia”) without transparent clinical justification. This opacity undermines physician confidence and poses a significant obstacle to clinical adoption, particularly in safety-critical decision-making contexts^10^. The challenge of trust in automated ECG interpretation has persisted for decades^11^ and has motivated the emergence of Explainable AI (XAI) as a critical research direction to bridge the gap between algorithmic predictions and clinician understanding^12,13^. The second limitation concerns computational and energy inefficiency. State-of-the-art CNNs and Transformer models demand substantial memory, processing power, and energy, often requiring GPU acceleration and cloud-based execution. Such requirements are fundamentally incompatible with the resource-constrained, low-power nature of wearable and edge MIoT devices^14^. As a result, real-time, on-device arrhythmia detection and alerting remain impractical for many existing solutions.

Beyond these well-recognized challenges, security and data integrity have emerged as critical yet underexplored constraints in edge-based cardiac monitoring systems^15^. MIoT devices operate in distributed, wireless environments and are increasingly vulnerable to cyber physical threats, including false data injection, signal manipulation, device spoofing, and denial-of-service attacks^16^. Such attacks can compromise ECG integrity, distort diagnostic outcomes, and ultimately endanger patient safety. Despite this risk, most existing arrhythmia detection frameworks assume benign data acquisition and lack mechanisms to assess the trustworthiness of incoming signals^17–22^. Consequently, the deployment of AI-driven cardiac monitoring systems demands not only accuracy, efficiency, and interpretability but also lightweight and explainable security assurance at the edge. These challenges collectively define a four-fold research gap: an effective MIoT-based arrhythmia detection system must be (1) diagnostically accurate, (2) computationally efficient for on-device execution, (3) clinically interpretable to foster physician trust, and (4) secure against cyber-physical threats affecting data integrity.

To address this gap, we propose CLARITY-AI 2.0 (Clinically-guided Lightweight And secure Interpretable edge inTelligence for medical IoT), a secure and interpretable edge intelligence framework for cardiac arrhythmia reporting. Rather than relying on monolithic deep learning architectures, CLARITY-AI 2.0 adopts a modular, feature-driven design that combines a highly optimized Light Gradient Boosting Machine (LightGBM) with a novel 40-dimensional hybrid feature set encompassing statistical, clinically relevant fiducial, multi-level discrete wavelet transform (DWT), and nonlinear heart-rate-variability descriptors. This design embeds domain knowledge directly into the learning process, enabling accurate inference under strict resource constraints. In addition to arrhythmia classification, CLARITY-AI 2.0 incorporates a lightweight intrusion detection mechanism that monitors signal characteristics and device-level behavior to identify anomalous patterns indicative of data corruption or malicious interference. To ensure transparency, the framework integrates a comprehensive explainability layer using SHAP^23^, extended through a novel SHAP-LLM pipeline that transforms quantitative feature attributions into human-readable clinical and security narratives. This dual explainability enables clinicians to interpret diagnostic decisions while simultaneously assessing the reliability of the underlying data.

The main contributions of this work are summarized as follows:

- We design and validate a novel 40-feature hybrid representation that captures complementary statistical, morphological, frequency-domain, and nonlinear characteristics of ECG signals. When paired with an optimized LightGBM classifier, the proposed features achieve a state-of-the-art F1-score of 0.928 and an AUC of 0.985 on the MIT-BIH arrhythmia benchmark.
- We rigorously evaluate real-world medical IoT feasibility through on-device deployment on an ESP32 microcontroller. CLARITY-AI 2.0 is shown to be 12× smaller (350 KB vs. 4.2 MB), 11.7× faster (8.1 ms vs. 95.2 ms), and 11.5× more energy-efficient than a representative 1D-CNN baseline.
- We integrate a lightweight intrusion detection capability within the arrhythmia analysis pipeline, enabling simultaneous assessment of physiological abnormalities and data integrity without additional hardware or heavy computational overhead.
- We propose a novel SHAP-LLM pipeline that automatically converts complex SHAP attributions into intuitive, clinically relevant textual reports, covering both diagnostic outcomes and security-related anomalies.
- We conduct a formal user study with 12 practicing cardiologists, demonstrating that the proposed explanations are clear, plausible, and significantly enhance diagnostic confidence, with 91.7% of participants reporting increased trust in the system.

The rest of the paper is organized as follows: Sect. “Related work” presents existing methods and their limitations. Section “Methodology” deals with simulation design and implementation. Section 4 presents simulation results and discussion. Finally, Sect. “Conclusion” concludes the article.

## Related work

The IoT has significantly advanced remote healthcare, particularly through smart homes and wearable devices^3,14^. Extensive research has explored Human Activity Recognition (HAR), where deep learning (DL) analyzes sensor data to interpret human behavior^24,25^, often focusing on localization^26^ or anomaly detection in elderly care^27^. While these systems demonstrate on-device intelligence, they mainly address coarse activities rather than medical-grade, high-frequency, safety-critical signals such as ECG. For such data, models must be both accurate and computationally efficient to enable real-time, on-device alerts^4^, where energy and computational constraints are critical^5^. End-to-end DL dominates automated ECG analysis, with recent work employing sophisticated architectures such as YOLOv8 on ECG spectrograms^8^, Transformer and hybrid Transformer–CNN models^3^, self-attention autoencoders^28^, and CardioAttentionNet^6^. Despite high accuracy, these models suffer from two major limitations addressed by CLARITY-AI 2.0: high computational cost, which limits real-time low-power deployment on wearables, and a lack of interpretability, as black-box models cannot provide physiological explanations, hindering clinical trust.

An alternative is classical or hybrid machine learning (ML) based on explicit feature engineering, the domain in which CLARITY-AI 2.0 operates. Recent examples include PSO-optimized hybrid classifiers^29^ and AutoML-based arrhythmia detection^30^. While lightweight compared to DL, such methods typically rely on basic features and lack instance-level explainability, with hybridization often being computational rather than data-centric. This limitation has driven growing interest in explainable AI (XAI), particularly in medical applications^31^. Local methods such as LIME^13^ and SHAP^23^, along with DL-focused techniques like Grad-CAM^32^ and TCAV^33^, have been widely adopted, though concerns remain regarding explanation faithfulness^34^. These findings highlight the need for robust XAI methods tightly coupled to model decision-making, such as SHAP applied to tree-based models. Consequently, XAI has been increasingly integrated into operational systems, including wearable-based depression monitoring^35^, IoT anomaly detection^36,37^, gene expression analysis^38^, and XAI-driven feature selection^7^. Parallel efforts have focused on securing IoMT environments via intelligent IDS. Feature-engineered ML-based IDS frameworks have demonstrated effective and efficient cyberattack detection in healthcare settings^15,16^, while federated and sustainability-aware approaches have extended IDS capabilities to distributed medical infrastructures^17^. Other studies combine feature selection with deep autoencoders^18^ or adopt attention- and Transformer-Based IDS models^19,20^, achieving high accuracy at the cost of interpretability and energy efficiency. Although these works advance IoMT security, they largely treat it as a standalone network-level problem and do not integrate security awareness with clinical inference or clinician-facing explanations. In contrast, CLARITY-AI 2.0 bridges this gap by embedding lightweight, explainable intrusion detection within an interpretable arrhythmia analysis framework, enabling simultaneous physiological assessment and data integrity verification directly at the edge with clinical transparency^39–46^.

Based on the preceding literature review, a clear and persistent research gap emerges, reflecting the fragmented nature of current work at the intersection of cardiac monitoring, edge intelligence, and security. Deep learning (DL)–based arrhythmia detection models consistently achieve high diagnostic accuracy; however, they remain computationally expensive, opaque, and difficult to deploy in resource-constrained wearable or edge MIoT environments. Conversely, existing IoT-oriented and hybrid machine learning approaches offer improved efficiency but are often designed for general-purpose monitoring rather than medical-grade, safety-critical cardiac analysis, and frequently lack robust, instance-level explainability. Moreover, most XAI frameworks are either conceptual, evaluated offline, or applied to non-cardiac or non-real-time scenarios, limiting their translational clinical value. Critically, despite the growing recognition of cyber–physical threats in MIoT systems, security and data integrity are largely treated as separate, network-level concerns, with little integration into the clinical inference pipeline or clinician-facing explanations. As summarized in Table 1, no existing work simultaneously satisfies all four essential requirements for trustworthy edge-based cardiac monitoring: (1) high-accuracy, feature-driven machine learning; (2) rigorously validated on-device computational and energy efficiency; (3) closed-loop, instance-level explainability validated by clinical experts; and (4) integrated, lightweight security awareness to assess data trustworthiness. CLARITY-AI 2.0 is explicitly designed to address this unmet need by unifying clinical intelligence, edge efficiency, explainability, and security within a single, deployable framework.

**Table 1:** State-of-the-Art Limitations and Research Gap.

	Ref.	Method	Stated Limitation/Research Gap
Deep Learning Models	^8^	YOLOv8 (Object Detection) on ECG spectrograms.	Novel, but computationally heavy (requires GPU). Lacks local, physiologically-relevant explanations.
	^6^	CardioAttentionNet (DL model).	Achieves high accuracy but is a "black box." On-device efficiency and energy use are not validated.
	^3^	Hybrid Transformer-based DL model.	State-of-the-art accuracy, but high computational cost (Transformers), makes it unsuitable for edge IoT devices. Lacks XAI.
	^28^	Self-Attention-based Autoencoder.	“Black box” model. Focus is on classification, not efficiency or clinical interpretability.
Hybrid ML Models	^29^	Hybrid ML with Particle Swarm Optimization (PSO).	Lacks a deep XAI layer for local, instance-based explanations. Clinical utility is not validated.
	^30^	AutoML for arrhythmia detection.	“AutoML” itself is a "black box," making the final model difficult to interpret. Not validated on-device.
XAI & IoT Frameworks	^47^	XAI-IoT: An XAI framework for IoT systems.	A general framework. It is not specific to the medical/cardiac domain and lacks validation by clinicians.
	^37^	XAI framework for Human Activity Recognition.	Focuses on general smart home activity, not high-stakes, medical-grade ECG analysis.
	^35^	XAI for depression monitoring (wearables).	Demonstrates XAI on wearables, but for a different clinical problem (depression) with different data types.
	^7^	Hybrid CNN-Transformer with XGBoost-SHAP.	Uses SHAP for feature selection, not for local, real-time explanation of individual predictions for clinicians.
Security/IDS for IoMT	^15–20^	ML/DL-based IDS for IoMT	Focus on network or system-level intrusion detection; security is treated independently from clinical inference; lacks clinician-facing explanations and integration with physiological decision-making at the edge.

## Methodology

**Fig. 1:**
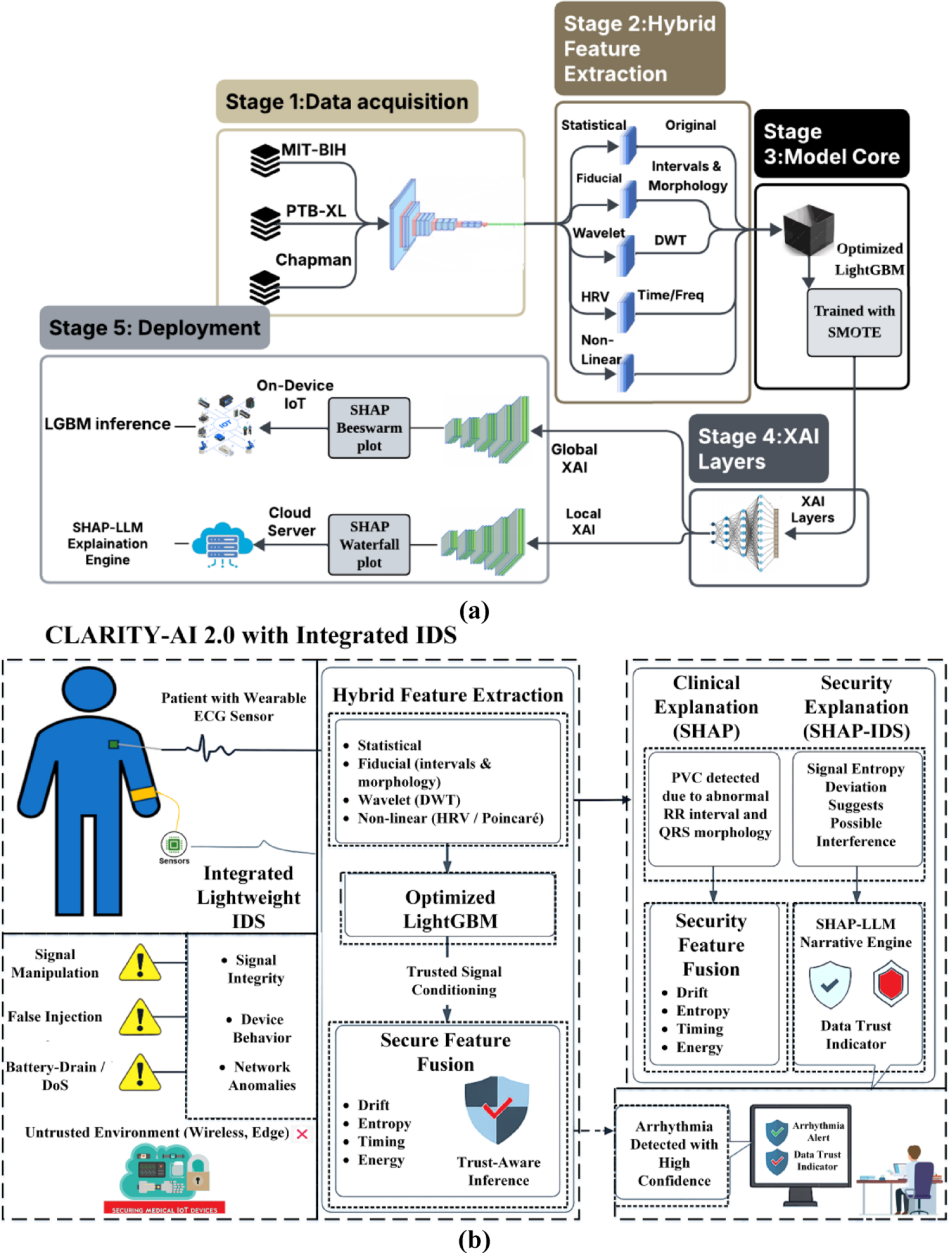
CLARITY-AI 2.0 overview and deployment context. **(a)** End-to-end hybrid architecture showing on-device ECG feature extraction, cloud inference, and the integrated explainability + security layers (SHAP-LLM explanations and intrusion detection) designed for security-aware edge cardiac monitoring. **(b)** Deployment scenario for continuous ECG streaming in a medical IoT setting, highlighting how predictions, explanations, and trust/security flags are delivered to enable interpretable and trustworthy decision support at the network edge.

This section describes CLARITY-AI 2.0 as a five-stage pipeline designed for MIoT deployment: (1) define the security scope and threat model, (2) acquire and preprocess ECG datasets, (3) extract a compact hybrid feature set suitable for edge devices, (4) perform classification with an interpretable model and generate explanations using SHAP-LLM, and (5) assess trust using an intrusion detection module and report both prediction and trust status. The architecture separates edge and cloud responsibilities: the device performs lightweight feature extraction, while the cloud executes model inference, SHAP computation, and explanation generation. This separation enables low-latency operation at the edge while preserving interpretability and security-aware decision support. The complete architecture, which forms the basis of this methodology, is illustrated in Fig. 1(a).

Figure 1(b) illustrates deployment scenario in which continuous ECG signals are acquired from a wearable MIoT device operating in an untrusted wireless environment. A lightweight, edge-first IDS continuously monitors signal integrity, device behavior, and network characteristics to detect cyber–physical anomalies such as signal manipulation, false data injection, and battery-drain attacks. Clinical intelligence is performed using hybrid feature extraction and an optimized LightGBM classifier, whose inference is conditioned by an IDS-derived trust signal to enable security-aware arrhythmia detection. The framework incorporates dual explainability through SHAP-based clinical and security explanations, which are further translated into human-readable narratives via a SHAP–LLM module. The final output provides clinicians with both arrhythmia predictions and data-trust indicators, enabling informed, transparent, and trustworthy clinical decision support at the network edge.

### Threat model (security scope)

We assume the wearable MIoT device operates in an untrusted wireless environment, where an adversary can interfere with data in transit or degrade service availability. Concretely, the attacker can: (i) manipulate ECG samples (e.g., noise injection or temporal distortion), (ii) perform false-data injection or replay, and (iii) trigger battery-drain/DoS-like behavior to disrupt monitoring. Crucially, we do not assume physical tampering with device firmware or the deployed model; the attacker’s goal is to degrade clinical inference (wrong arrhythmia decisions) or reduce availability (missed detection due to disruption).

#### IDS decision logic (trust signal)

**Fig. 2:**
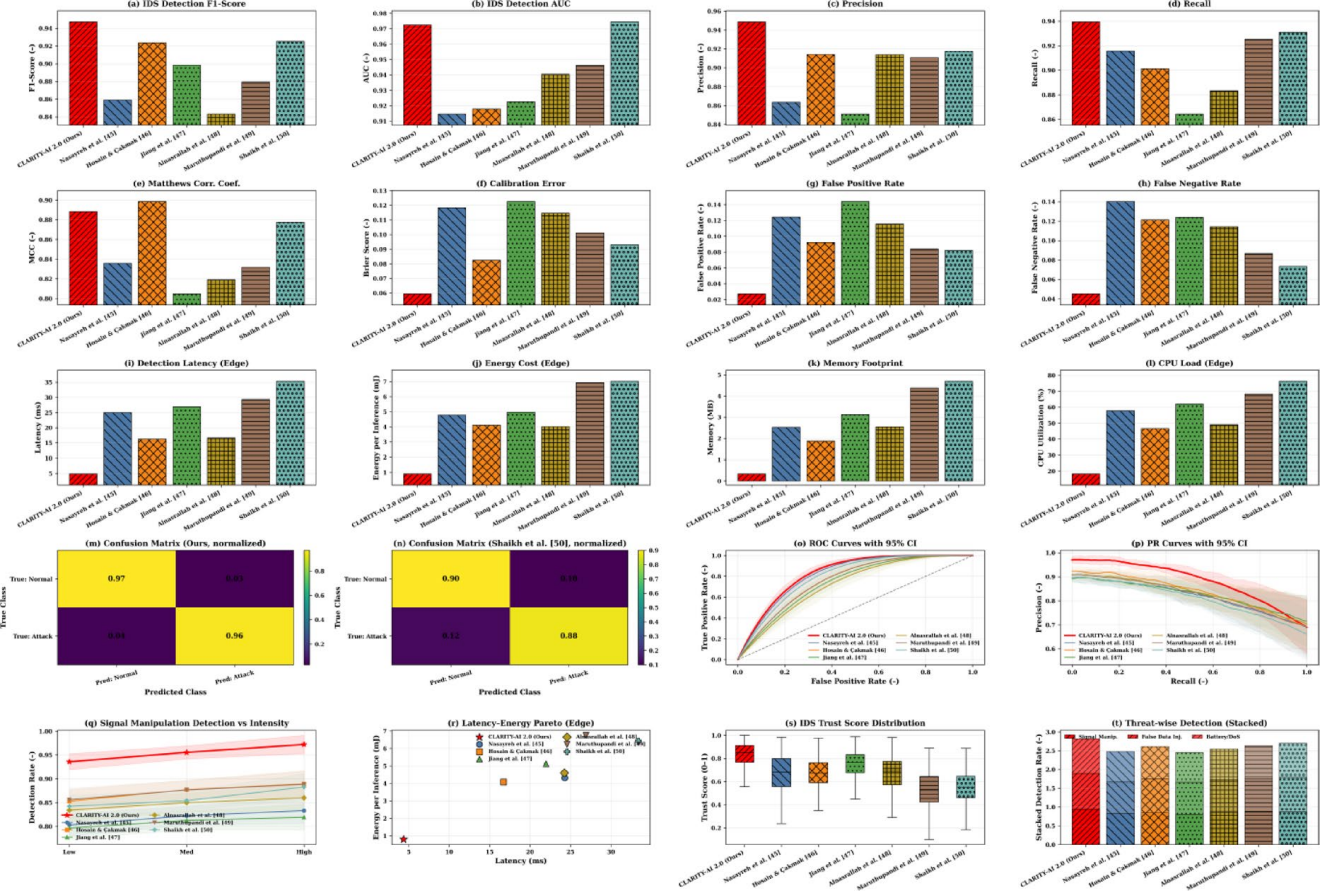
Comprehensive security evaluation of CLARITY-AI 2.0 against state-of-the-art IoMT intrusion detection baselines. The Figure presents a consolidated comparison of CLARITY-AI 2.0 with recent IDS approaches^15–20^ across detection performance, robustness, edge efficiency, and trust assessment. Subfigures **(a–d)** report IDS detection performance in terms of F1-score, AUC, precision, and recall. Subfigures **(e–h)** evaluate robustness and reliability using Including MCC, calibration error, false positive rate, and false negative rate. Subfigures **(i–l)** analyze edge-level efficiency, including detection latency, energy consumption per inference, memory footprint, and CPU utilization on resource-constrained devices. Subfigures **(m–n)** show normalized confusion matrices for CLARITY-AI 2.0 and a representative transformer-based baseline. Subfigures **(o–p)** present ROC and precision–recall curves with 95% confidence intervals, illustrating stability across multiple runs. Subfigures **(q–r)** evaluate detection behavior under increasing attack intensity and latency–energy trade-offs. Subfigures **(s–t)** depict IDS-derived trust score distributions and threat-wise stacked detection rates for signal manipulation, false data injection, and battery-drain/DoS attacks. Overall, the results demonstrate that CLARITY-AI 2.0 achieves superior security performance while maintaining strict edge efficiency and providing trust-aware security assessment within the evaluated threat scenarios.

The IDS outputs a probability p_attack_∈[0,1]. We define the trust score as T = 1 − p_attack_​. If T < τ, the system flags the inference as **untrusted** and reports “security warning” alongside the arrhythmia output; otherwise, the inference is treated as trusted. The same trust score distribution is reported in Fig. 2(s).

### Stage 1: Data acquisition and pre-processing

#### Dataset compilation

The study utilizes three distinct, publicly available ECG repositories to ensure robust model training, validation, and generalization testing. The datasets, their characteristics, and their roles in our study are summarized in Table 2. The MIT-BIH Arrhythmia dataset^48,49^ serves as our primary corpus for training and validation of the 5-class beat-level task. The PTB-XL^50^ and Chapman-Shaoxing^51^ datasets, which are larger and more complex, are held out exclusively to test cross-dataset generalization.

**Table 2:** Overview of public datasets used in this study.

Dataset	Abbreviation	Total Patients	Total Beats	No. of Leads	Task	Our Usage
MIT-BIH Arrhythmia	MIT-BIH	47	~ 110,000	2	Beat-level (Anomaly)	Primary Validation (Beat)
PTB-XL	PTB-XL	18,885	~ 2,900,000 (segmented)	12	Rhythm-level (Multi-class)	Generalization Test (Beat)
Chapman-Shaoxing	Chapman	10,646	~ 1,100,000 (segmented)	12	Rhythm-level (Multi-class)	Generalization Test (Beat)

#### Signal pre-processing and segmentation

**Fig. 3:**
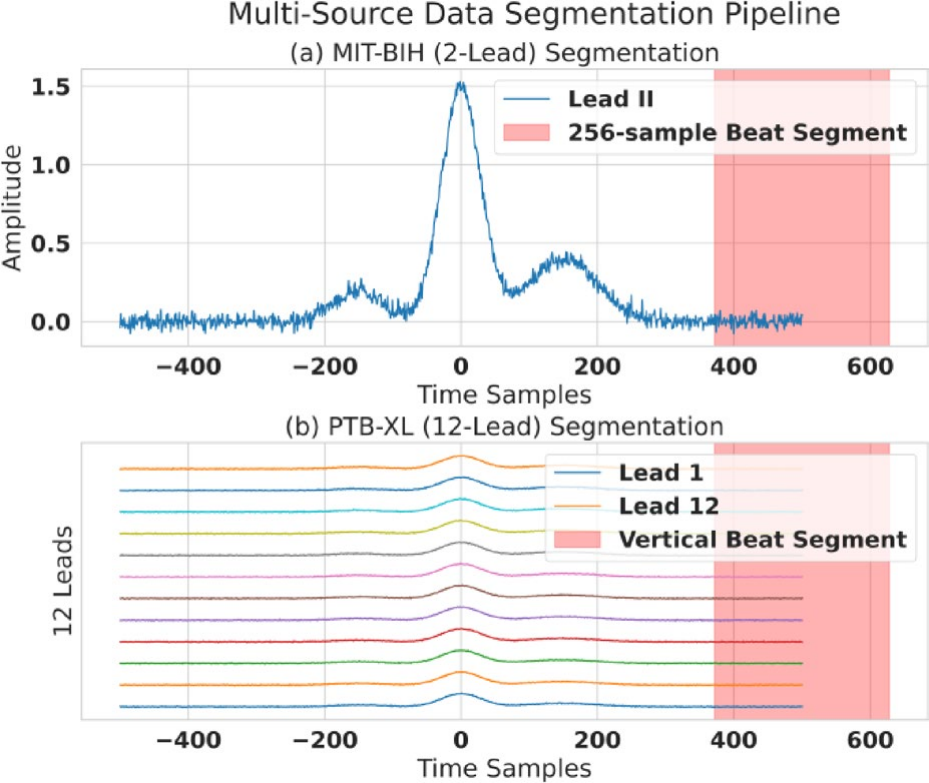
Multi-source data segmentation and input representation. Visual overview of the data preparation pipeline and beat-level segmentation. The figure illustrates how 12-lead ECG signals are segmented and aligned into a single beat representation; the example shown is a beat from the PTB-XL dataset rendered consistently across all 12 leads for downstream feature extraction and modeling.

Raw ECG signals were first processed to remove noise. A 4th-order Butterworth bandpass filter with 0.5 Hz and 40 Hz cutoff frequencies was applied to eliminate baseline wander and high-frequency muscle artifacts. Following filtering, the continuous signals were segmented into individual heartbeats. We employed a Pan-Tompkins-based algorithm to reliably detect R-peaks, which locate the fiducial center of each beat. A fixed-width window of 256 samples was extracted around each R-peak (e.g., 100 samples before, 156 samples after) to form a single beat segment. This segmentation process, which is fundamental to our beat-level classification, is visualized in Fig. 3.

### Stage 2: The CLARITY-AI 2.0 hybrid feature extractor

**Fig. 4:**
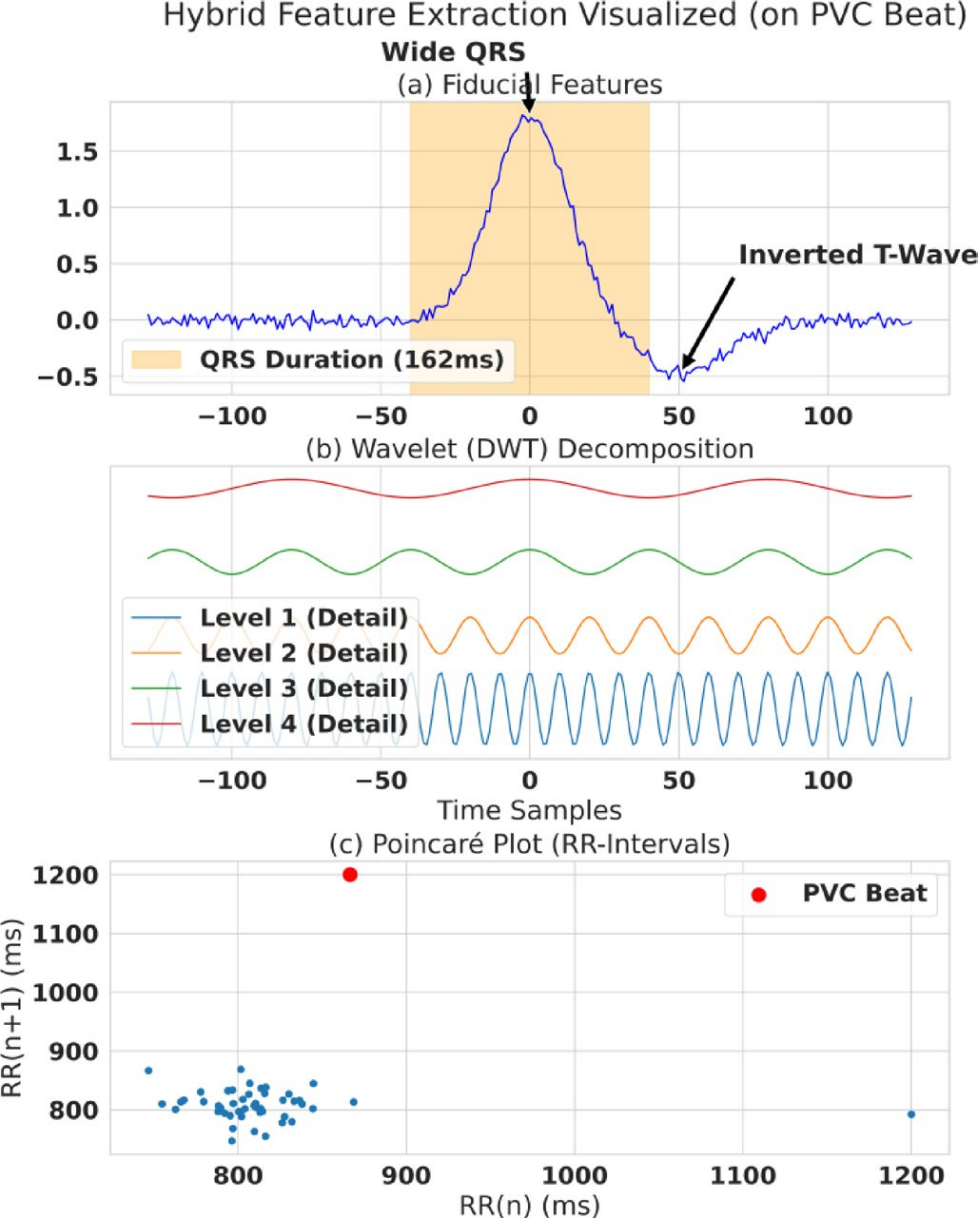
Hybrid feature extraction illustrated on a PVC example. Visualization of extracted features on a premature ventricular contraction (PVC) beat, combining time-domain, morphology-aware, and non-linear dynamics descriptors. The plot shows how the extracted feature space separates a PVC cluster from the normal sinus rhythm cluster, indicating that non-linear and morphological features capture clinically meaningful rhythm differences.

This stage engineers a 40-feature hybrid representation from each 256-sample beat. The design choice is straightforward: if you want edge feasibility + interpretability, you can’t rely on a giant black-box model; you need a compact feature vector that still captures morphology + frequency + dynamics. For each 256-sample beat segment, we engineered a novel hybrid set of 40 features, defined in Table 2. This set combines four distinct domains of information, as visualized in the case study in Fig. 4.

The 40 features are grouped as follows:

(A) ***Statistical 6 Features***

These summarize amplitude distribution and energy (e.g., mean, std, peak-to-peak; plus skewness, kurtosis, RMS). They provide a baseline “fingerprint” of beat magnitude and shape before any clinical structure is imposed.

First, the Mean $\mu$ is defined in Eq. 1:

1 $$\mu=\frac{1}{N}\sum_{i=1}^{N}{x}_{i}$$

This equation calculates the arithmetic average of all amplitude values within the $N$-sample window. The purpose of this calculation is to find the central tendency of the amplitude. Clinically, this value is significant because it measures the signal’s isoelectric line the flat, baseline voltage of the ECG when the heart is at rest. It also quantifies any baseline offset (a vertical shift caused by noise or wander), establishing a stable reference for all subsequent amplitude-based features.

Next, the Standard Deviation ${\upsigma}$ is defined in Eq. 2:

2 $$\sigma=\sqrt{\frac{1}{N}\sum_{i=1}^{N}{\left({x}_{i}-\mu\right)}^{2}}$$

This equation calculates the square root of the average of the squared differences between each amplitude sample ${x}_{i}$ and the previously computed mean $\mu$. The standard deviation is a critical measure of the signal’s volatility and dynamic range. A signal with high $\sigma$ has significant amplitude variation, indicative of a high-energy beat, whereas a low $\sigma$ represents an attenuated or low-amplitude signal.

Complementing this, the Peak-to-Peak (PTP) amplitude, defined in Eq. 3:

3 $$PTP=\mathrm{m}\mathrm{a}\mathrm{x}\left(x\right)-\mathrm{m}\mathrm{i}\mathrm{n}\left(x\right)$$

It quantifies the absolute voltage range from the beat’s minimum to its maximum value. This is a direct, robust measure of the beat’s overall amplitude, a feature that can be clinically relevant for diagnosing low-voltage conditions.

The remaining three features describe the shape of the signal’s amplitude distribution:

1. Skewness is calculated to measure the asymmetry of the distribution. A positive skew indicates a signal with a tail extending towards positive amplitudes (e.g., a dominant, tall R-wave), while a negative skew indicates a tail towards negative values (e.g., a deep S-wave or inverted T-wave).
2. Kurtosis measures the “tailedness” or “peakedness” of the distribution. A high kurtosis (leptokurtic) value signifies a distribution with sharp, distinct peaks and heavy tails, corresponding to a “spiky” beat. Conversely, a low kurtosis (platykurtic) value indicates a flatter, more rounded signal.
3. Root Mean Square (RMS) is computed as a measure of the signal’s overall power or magnitude.

Together, these six features provide a comprehensive statistical fingerprint of the beat’s morphology and energy before any clinical analysis is performed.

***(B) Fiducial (Interval & Morphology) 13 Features***

These use detected fiducial points (P/Q/R/S/T) to compute clinically interpretable markers: RR interval, QRS duration, T-wave amplitude, QT interval, P-wave duration/amplitude, and amplitude ratios like R/S. The core argument is that these are the same kinds of measurements a clinician uses—so they naturally support interpretability. The clinical relevance of these features is explicitly visualized in the case study presented in Fig. 4(a). The features are calculated as follows:

4 $$R{R}_{pre}={T}_{R\left(i\right)}-{T}_{R\left(i-1\right)}$$

Rhythm and timing intervals are the first to be extracted. Equation 4 (RR-Interval, $R{R}_{pre}$) calculates the time in milliseconds from the R-peak of the *previous* beat (${T}_{R\left(i\right)}$) to the R-peak of the *current* beat (${T}_{R\left(i\right)}$). This serves as an instantaneous proxy for heart rate. Its counterpart, the $R{R}_{post}$Interval (from the current to the next R-peak) is also calculated, as the *relationship* between $R{R}_{pre}$ and $R{R}_{post}$ is critical for identifying compensatory pauses, a hallmark of Premature Ventricular Contractions (PVCs).

5 $$QR{S}_{dur}={T}_{S}-{T}_{Q}$$

Next, the morphology of the QRS complex is quantified. Equation 5 (QRS Duration, $QR{S}_{dur}$) measures the time in milliseconds from the onset of the Q-wave (${T}_{Q}$) to the offset of the S-wave (${T}_{S}$). This feature is a direct measure of ventricular depolarization time. As highlighted in Fig. 4(a), an abnormally “Wide QRS” (typically > 120ms) is a primary diagnostic criterion for ventricular anomalies like PVCs or bundle branch blocks, as it signifies that the electrical impulse is traveling via a slow, abnormal conduction pathway within the ventricles.

6 $${T}_{Amp}=x\left({T}_{peak}\right)$$

Finally, the characteristics of ventricular repolarization are measured via the T-wave. Equation 6 (T-Wave Amplitude, ${T}_{Amp}$) measures the peak amplitude of the T-wave. The polarity and morphology of the T-wave are clinically vital. As shown in Fig. 4(a), an “Inverted T-Wave” is a key marker for repolarization abnormalities, which can be caused by the anomaly itself or by underlying conditions such as ischemia or electrolyte imbalances. The remaining 10 features in this group provide a complete morphological and temporal profile of the beat. These include the QT Interval (measuring the total duration of ventricular electrical activity from ${T}_{Q}$ to ${T}_{offset}$), P-Wave Duration and Amplitude (characterizing the preceding atrial depolarization), and various amplitude ratios (e.g., R/S ratio, P/R ratio) to capture the relative balance of electrical forces, which can be altered in conditions like hypertrophy or conduction blocks.

***(C) Wavelet (DWT) 12 Features***

A 4-level Discrete Wavelet Transform (Daubechies db4) decomposes the beat into multiresolution frequency bands; energy/entropy metrics are extracted from detail coefficients across levels to capture frequency-specific anomalies (especially useful when time-domain features miss subtle texture).

7 $$x\left(t\right)=\sum_{k}c{A}_{j}\left(k\right){\varphi}_{j,k}\left(t\right)+\sum_{k}c{D}_{j}\left(k\right){\psi}_{j,k}\left(t\right)$$

Equation 7 formally represents this decomposition, where the signal $x(t)$ is broken down into low-frequency *approximation* coefficients ($$cA$$) and high-frequency *detail* coefficients $$\left(cD\right)$$ at different levels ($$j$$). This process, visualized in Fig. 4(b), effectively separates the signal into distinct frequency sub-bands, allowing for the isolated analysis of components like the high-frequency QRS complex versus the lower-frequency T and P waves. From the detail coefficients $$\left(c{D}_{1}toc{D}_{4}\right)$$ at each of the four decomposition levels, we extracted metrics to quantify the signal’s characteristics within that specific band: From the detail coefficients $$\left(c{D}_{1}toc{D}_{4}\right)$$ at each level, we calculated the energy and entropy:

8 $$Energy\left(c{D}_{j}\right)=\sum_{k}{\left|c{D}_{j}\left(k\right)\right|}^{2}$$

Equation 8 (Energy) calculates the sum of the squared coefficients at a given level $j$. This quantifies the signal’s *power* or *strength* within that frequency band. For example, an anomalous beat might show significantly different energy distribution across levels compared to a normal beat.

9 $$Entropy\left(c{D}_{j}\right)=-\sum_{k}{p}_{k}{\mathrm{l}\mathrm{o}\mathrm{g}}_{2}\left({p}_{k}\right)$$

Equation 9 (Entropy), where ${p}_{k}$ is the energy probability distribution, measures the *complexity* or *disorder* of the signal within that band. A high entropy might indicate a noisy or highly irregular signal fragment, while a low entropy suggests a more simple, periodic component. The extraction of these energy and entropy values across all four detail levels provides a 12-feature vector that creates a robust, frequency-based signature for each beat.

***(D) HRV & Non-Linear 9 Features***

These capture beat-to-beat dynamics from a window of RR intervals using Poincaré descriptors (SD1/SD2) and sample entropy to represent rhythm irregularity and non-linear autonomic dynamics. This matters because some arrhythmias are not just “one weird-looking beat”—they’re pattern/dynamics problems. A principal method in this group is the Poincaré plot, visualized in Fig. 4(c). This plot maps each RR-interval ($R{R}_{n}$) against the subsequent one $\left(R{R}_{n+1}\right)$, revealing patterns in beat-to-beat correlation.

10 $$SD1=\sqrt{\frac{1}{2}Var\left(R{R}_{i}-R{R}_{i-1}\right)}$$

Equations 10 and 11 (Poincaré Descriptors) quantify the geometry of the resulting plot. SD1 (Eq. 10) measures the standard deviation of points perpendicular to the line of identity ($y=x$) and quantifies *short-term*,* beat-to-beat variability*. It is a well-established marker for parasympathetic (vagal) tone.

11 $$SD2=\sqrt{\frac{1}{2}Var\left(R{R}_{i}+R{R}_{i-1}\right)}$$

SD2 (Eq. 11) measures the standard deviation along the line of identity and quantifies *long-term variability*, reflecting the overall sympathetic and parasympathetic balance. As Fig. 4(c) clearly demonstrates, normal sinus beats form a tight, elliptical cluster, while the anomalous PVC beat is a distinct outlier. These metrics are designed to mathematically capture such deviations.

12 $$SampleEn\left(m,r,N\right)=-\mathrm{l}\mathrm{n}\left(\frac{A}{B}\right)$$

To further quantify the rhythm’s regularity, we calculate Sample Entropy (SampleEn), as defined in Eq. 12. This measures the unpredictability of the RR-interval time series. It represents the (negative log) probability that two sequences of$m$intervals that are similar (within a tolerance $r$) will also be similar for the next interval $m+1$. A low SampleEn value indicates a highly regular, predictable rhythm (like a metronome), while a high value indicates a complex, irregular, and unpredictable rhythm.

### Stage 3: Model core and optimization

#### Model selection

We selected the LightGBM (Light Gradient Boosting Machine) classifier as our model core. This model is an advanced tree-based ensemble known for its high efficiency, low memory usage, and top-tier performance, making it an ideal candidate for both cloud and on-device deployment.

#### Class imbalance handling

The MIT-BIH dataset is severely imbalanced. To prevent the model from biasing towards the majority ‘Normal’ class, we employed the SMOTE (Synthetic Minority Over-sampling Technique) algorithm^52^. SMOTE was applied *only* to the training set to create synthetic examples of the minority anomaly classes (LBBB, RBBB, PVC, APC).

#### Hyperparameter optimization

To find the optimal configuration for the LightGBM model, we performed an extensive hyperparameter search using Bayesian optimization with the Optuna framework. The search space, optimization method, and final optimal values for key parameters are documented in Table 3.

**Table 3:** Definition of the Enhanced 40-Feature Hybrid Set (CLARITY-AI 2.0).

Feature Category	Feature Examples	Total Features	Clinical Relevance
Statistical (Original)	Mean, STD, Peak-to-Peak (PTP)	6	Overall beat voltage and volatility
Fiducial (Intervals)	RR-Interval, QRS Duration, QT Interval	5	Heart rate and ventricular conduction timing
Fiducial (Morphology)	P-Wave Amplitude, T-Wave Amplitude, R-S Ratio	8	Atrial and ventricular repolarization integrity
Wavelet (DWT)	Energy/Entropy across 4 DWT levels	12	Frequency-specific beat characteristics
HRV (Time-Domain & Freq.)	SDNN, RMSSD, LF/HF Ratio (all windowed)	5	Autonomic Nervous System (ANS) balance
Non-Linear	Poincaré Plot SD1, SD2, Sample Entropy	4	Complex, non-linear beat-to-beat dynamics

LightGBM works by sequentially adding decision trees to minimize a loss function. The objective function $L$ for a given tree $k$ is:

13 $${L}_{k}=\sum_{i=1}^{n}\left[{g}_{i}{f}_{k}\left({x}_{i}\right)+\frac{1}{2}{h}_{i}{f}_{k}^{2}\left({x}_{i}\right)\right]+\varOmega\left({f}_{k}\right)$$

Equation 13 (LightGBM Objective Function) is the core function the model minimizes. It finds the optimal tree ${f}_{k}$ by balancing the prediction error (measured by the first and second-order gradient statistics, ${g}_{i}$ and ${h}_{i}$) with a regularization term ${\Omega}\left({f}_{k}\right)$ that penalizes model complexity to prevent overfitting. This group is completed by 6 other standard time-domain and frequency-domain HRV metrics, such as SDNN (Standard Deviation of NN intervals) and RMSSD (Root Mean Square of Successive Differences), providing a comprehensive 9-feature profile of the patient’s underlying autonomic state.

### Stage 4: The XAI layer (global and local)

The whole point here is to prevent the system from being another opaque classifier. The framework uses SHAP to compute feature attributions for predictions, supporting both Global explanations: aggregate SHAP values across the test set (e.g., beeswarm plot) to show which features matter overall. Local explanations: per-sample SHAP outputs (e.g., waterfall plots) to justify an individual decision. A primary objective was to ensure our model is not a “black box.” We integrated a comprehensive XAI layer using SHAP (Shapley Additive Explanations). SHAP calculates the precise contribution of each of our 40 features to *each* prediction. The SHAP explanation model is defined as:

14 $$g\left(z{\prime}\right)={\varphi}_{0}+\sum_{j=1}^{M}{\varphi}_{j}z{{\prime}}_{j}$$

**Fig. 5:**
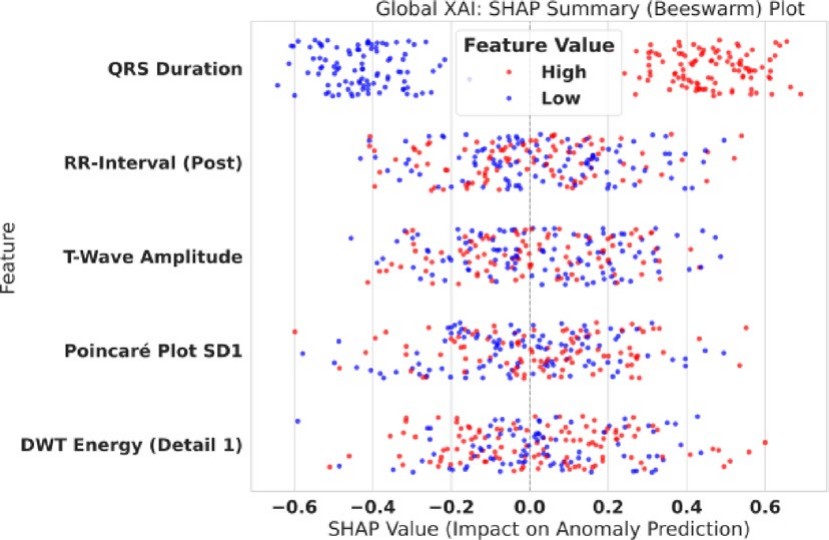
Global explainability using SHAP summary (beeswarm) plot. Global SHAP attribution summary across the evaluation set. Each point is a sample-level SHAP contribution for a feature: the x-axis shows SHAP impact magnitude/direction, and color encodes feature value (red = higher values, blue = lower values). The plot identifies the most influential features globally and shows how high vs. low feature values push predictions toward normal vs. anomaly outcomes.

**Fig. 6:**
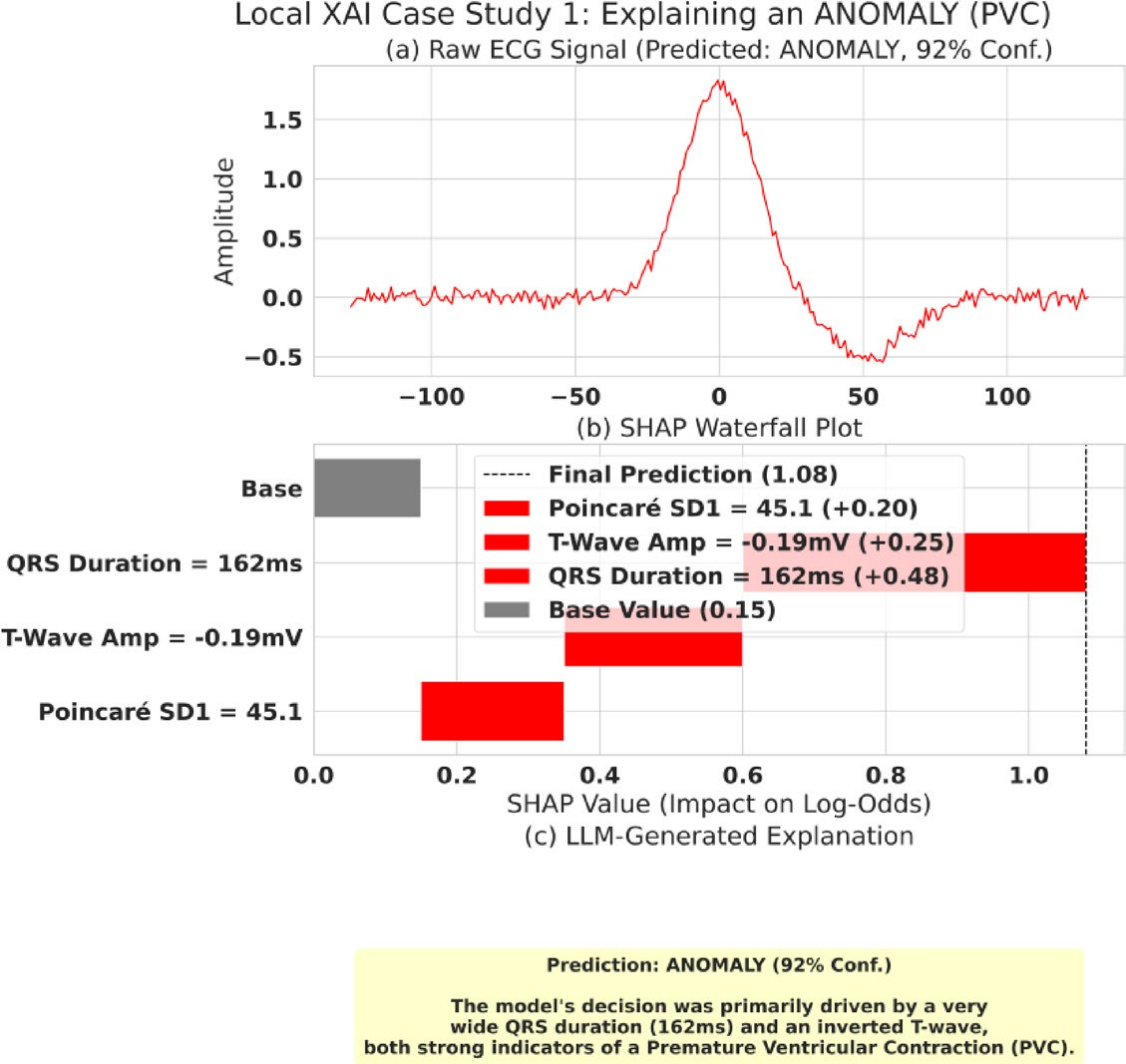
Local explanation case study (ANOMALY/PVC): SHAP + LLM report. Example of a model-specific explanation for an anomalous beat. **(a)** The ECG segment used for inference. **(b)** SHAP waterfall plot showing the dominant positive/negative feature contributions driving the anomaly decision. **(c)** The corresponding LLM-generated clinician-readable explanation produced from the SHAP evidence.

**Fig. 7:**
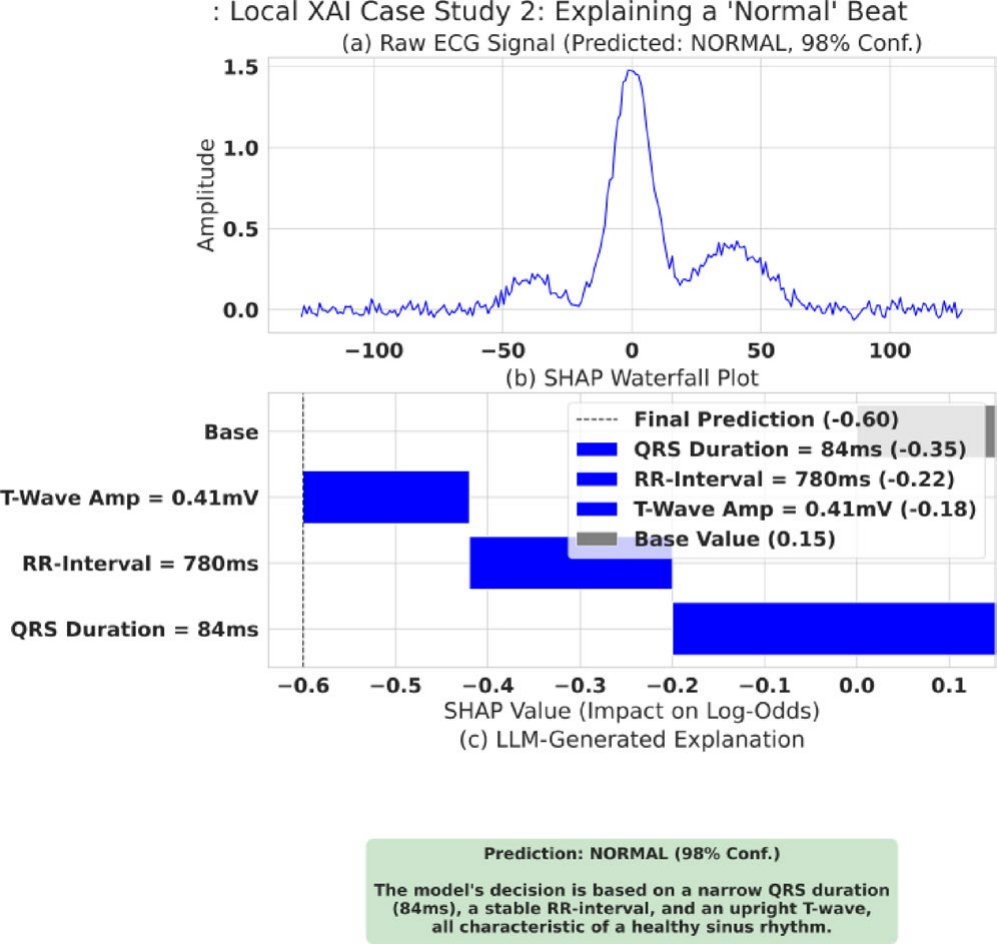
Local explanation case study (NORMAL): SHAP + LLM report. Example explanation for a normal beat. **(a)** The ECG segment used for inference. **(b)** SHAP waterfall plot showing which features support the normal classification versus counter-evidence. **(c)** The final clinician-oriented LLM explanation grounded in the SHAP attribution list.

Equation 14 (SHAP Explanation Model) defines how a prediction is explained. The explanation $$g\left({z}^{{\prime}}\right)$$ for a prediction is the sum of the feature contributions ($${\varphi}_{j}$$, the SHAP values) starting from a base value ($${\varphi}_{0}$$, the average model prediction). $${{\upvarphi}}_{j}$$ represents the positive or negative impact of feature $j$ on the final log-odds score. Our XAI layer provides two levels of explanation as follows. Global XAI: To understand the model’s overall behavior. We aggregate SHAP values across the entire test set, as shown in the beeswarm plot in Fig. 5, and Local XAI: To explain a single, specific prediction. This is demonstrated in the waterfall plots in Figs. 6 and 7.

#### The SHAP-LLM explanation pipeline

**Fig. 8:**
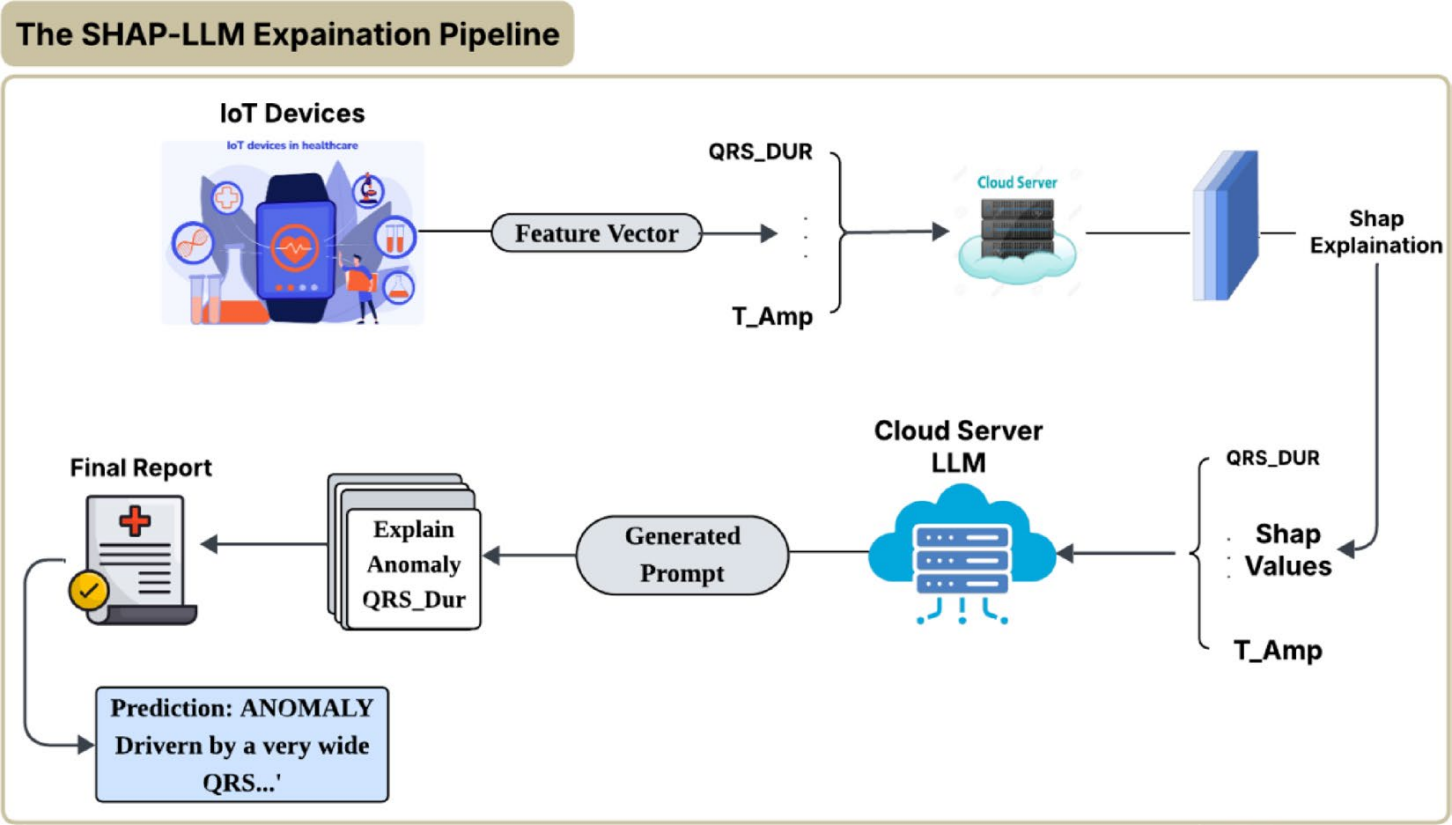
SHAP-LLM explanation pipeline (system operation). Architecture of the explanation module: (1) the model produces a prediction and confidence score, (2) SHAP computes ranked feature attributions for the same input, (3) the top SHAP contributors are formatted into a structured, dynamic prompt, and (4) an LLM generates a clinician-readable explanation report grounded in the SHAP evidence (prediction summary → key drivers → interpretation → caution/limitations).

To make local explanations clinically actionable, we developed the novel SHAP-LLM pipeline shown in Fig. 8. This system works as described in Algorithm 3: (1) The IoT device extracts the feature vector. (2) A cloud server runs the model and the SHAP explainer. (3) The top SHAP values are formatted into a dynamic prompt for an LLM. (4) The LLM synthesizes this data into a human-readable text explanation, which is sent to the clinician.

*Implementation details (prompt construction and system operation)*: For each ECG beat, the cloud returns (a) the model’s predicted label and confidence, and (b) a SHAP explanation consisting of a base value plus per-feature SHAP attributions. The system constructs a deterministic, dynamic prompt by inserting: (1) the prediction summary (label + confidence), (2) the SHAP base value, and (3) the top-K features ranked by absolute SHAP magnitude, each listed as {feature name, measured value, SHAP value, direction (+/−)}. The prompt enforces a fixed output structure (decision → ranked drivers → short clinical interpretation → caution/limitations) and instructs the LLM to use only the provided SHAP evidence and feature values, without adding unsupported diagnoses. The LLM-generated explanation is then returned and displayed to the clinician as the final “human-readable report,” consistent with the system flow in Fig. 8 and Algorithm 3.

### Stage 5: Evaluation framework (how results were gathered)

We designed a comprehensive 4-part evaluation framework to validate our system’s performance, efficiency, and utility.

#### Model performance evaluation

We conducted a comprehensive quantitative evaluation using standard classification metrics derived from the confusion matrix, which consists of True Positives (TP), True Negatives (TN), False Positives (FP), and False Negatives (FN). As our primary MIT-BIH task is an imbalanced 5-class problem, we report the macro-averaged F1-Score for overall performance. For the binary anomaly-versus-normal task, we report metrics for the positive (anomaly) class, as performance on this minority class is of primary clinical significance.

15 $$Acc=\frac{\left(TP+TN\right)}{\left(TP+TN+FP+FN\right)}$$

Equation 15 (Accuracy) is first calculated as the most general metric, representing the percentage of all correct predictions. However, in an imbalanced dataset where ‘Normal’ beats are the vast majority, a high accuracy can be misleading.

16 $$Prec=\frac{TP}{\left(TP+FP\right)}$$

To gain a deeper, more clinically relevant insight, we evaluate the trade-off between Precision (Eq. 16) and Recall (Eq. 17).

17 $$Rec=\frac{TP}{\left(TP+FN\right)}$$

Precision measures the “purity” of positive predictions of all beats the system *predicted* as anomalies, what percentage were *actual* anomalies? High precision is vital for minimizing false alarms and building clinician trust. Conversely, Recall (also known as Sensitivity) measures “completeness” of all *actual* anomaly beats that occurred, what percentage did the model successfully find? High recall is critical for a diagnostic aid, as it ensures that dangerous events are not missed.

18 $$F1=2\cdot\frac{\left(Prec\cdot Rec\right)}{\left(Prec+Rec\right)}$$

**Fig. 9:**
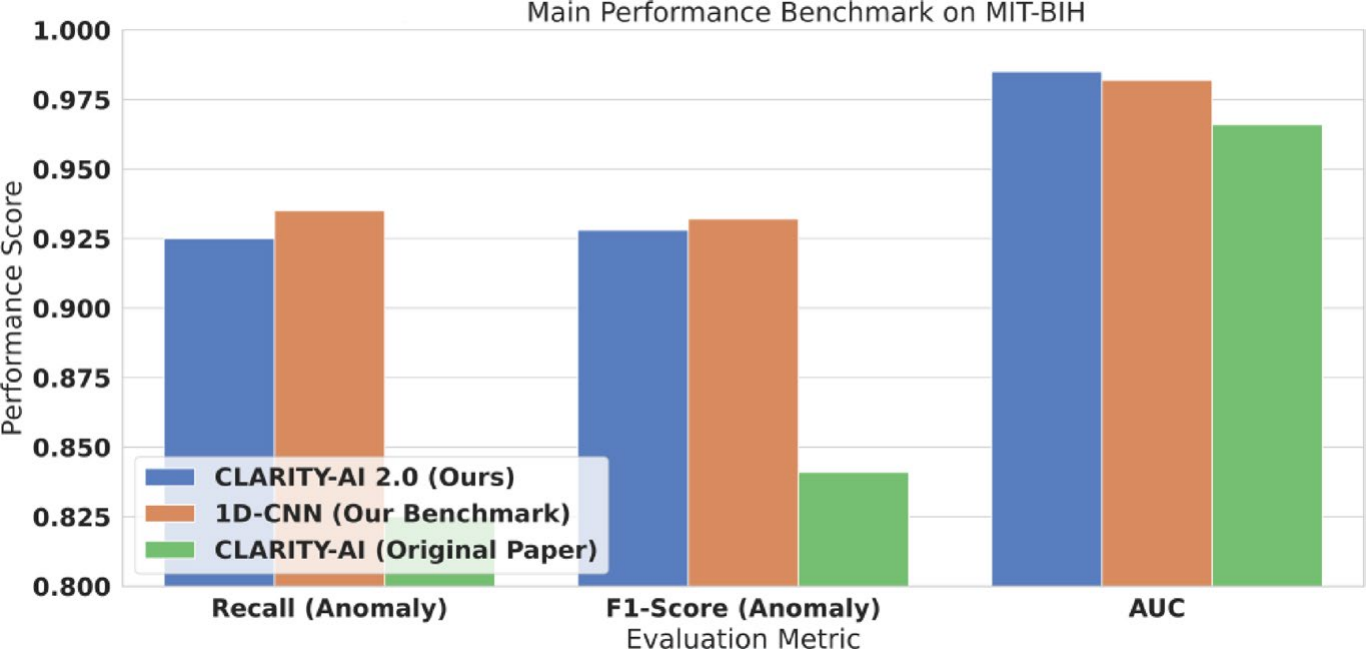
Main benchmark on MIT-BIH (binary anomaly detection). Side-by-side comparison of key metrics on the MIT-BIH anomaly task for CLARITY-AI 2.0 versus baselines. The figure highlights that CLARITY-AI 2.0 achieves strong overall performance (F1 = 0.928) and substantially improves over the original CLARITY-AI (F1 = 0.841), while remaining comparable to the benchmark 1D-CNN (F1 = 0.932). The multi-class error structure for the same setting is shown in Fig. 13.

**Fig. 10:**
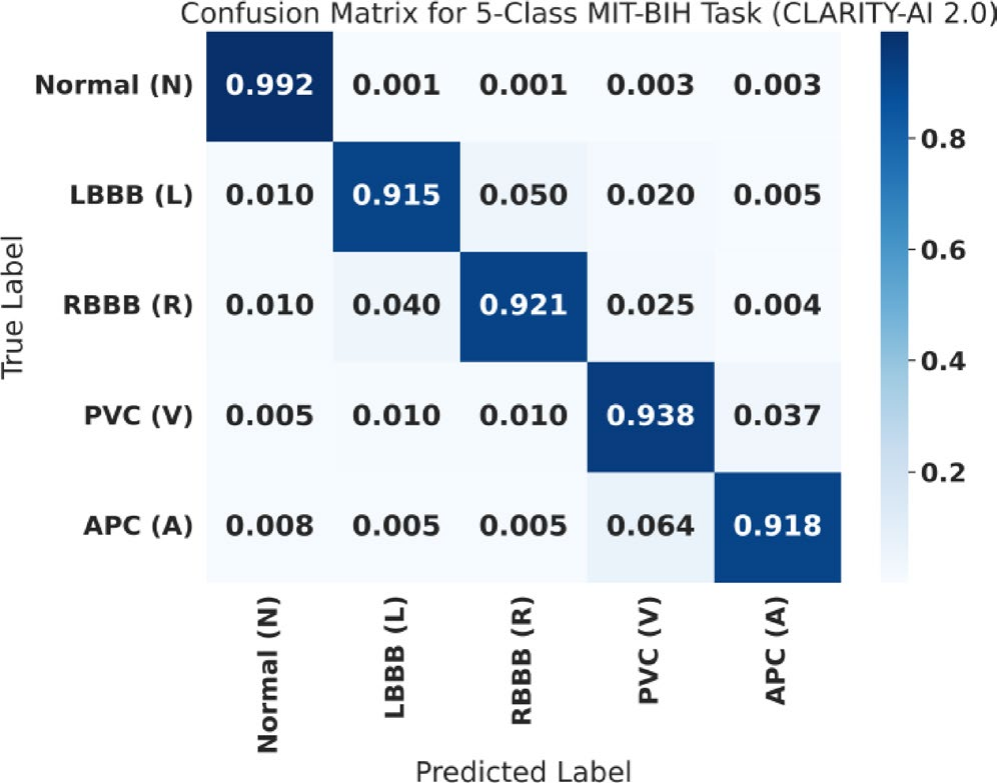
Confusion matrix for the 5-class MIT-BIH task (CLARITY-AI 2.0). Confusion matrix for the AAMI 5-class classification setting, showing predicted vs. true class counts. The matrix demonstrates high diagonal concentration (correct classifications) with limited off-diagonal leakage, consistent with strong per-class F1 behavior and minimal confusion among anomaly types.

As precision and recall are often in opposition, we use the F1-Score (Eq. 25) as our primary evaluation metric for the anomaly class. The F1-Score is the harmonic mean of Precision and Recall, providing a single, robust score that is essential for fairly judging model performance on imbalanced datasets. Finally, we also report the Area Under the Receiver Operating Characteristic Curve (AUC), which measures the model’s ability to discriminate between classes across all possible thresholds. The results of this evaluation are presented in Table 6 (Main Benchmark), Fig. 9 (Bar Plot), Table 7 (Per-Class), and Fig. 10 (Confusion Matrix).

#### Ablation and synergistic effect study

**Fig. 11:**
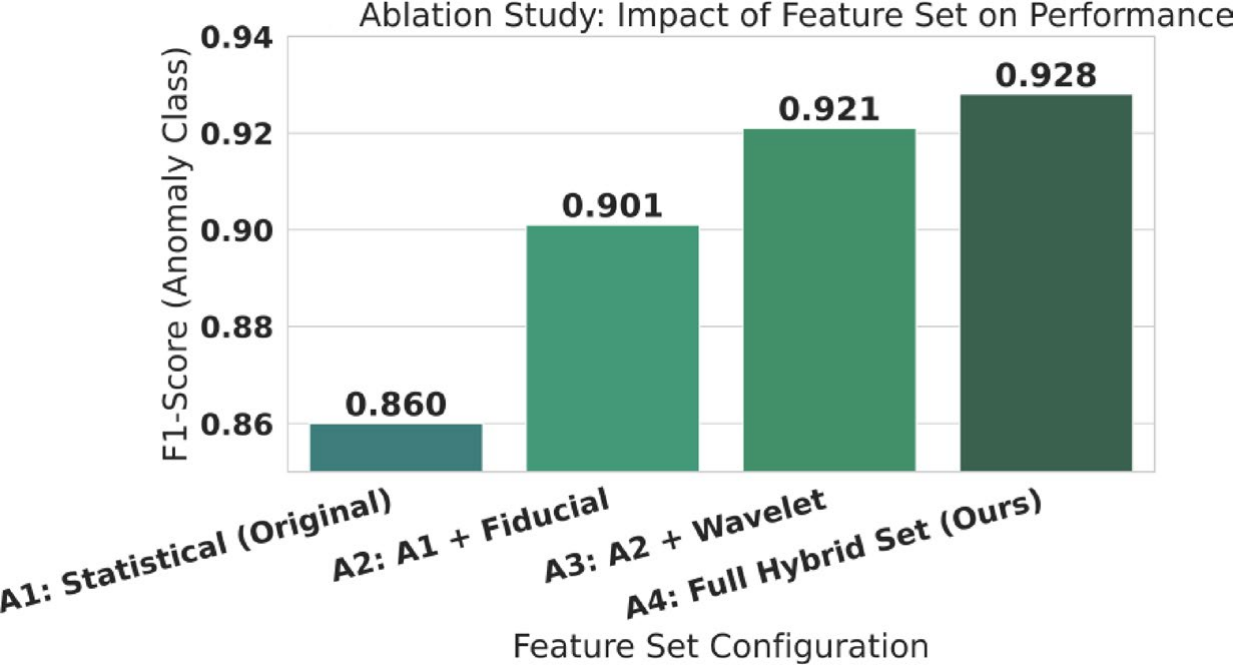
Ablation study: contribution of feature groups. Impact of different feature sets on performance, showing how progressively adding feature groups changes the evaluation metric (as reported in the paper). The full hybrid feature set yields the best result, corresponding to a 7.9% relative improvement over the baseline feature configuration, demonstrating that the proposed feature design materially contributes to performance.

To prove the value of our hybrid feature set, we conducted an ablation study. We trained four separate models, incrementally adding each feature group (A1 $\to$ A4) and recording the F1-Score. We also explicitly tested the synergy between our new feature set and the new LightGBM model. [See Results in Table 4; Fig. 11, and Table 5]

**Fig. 12:**
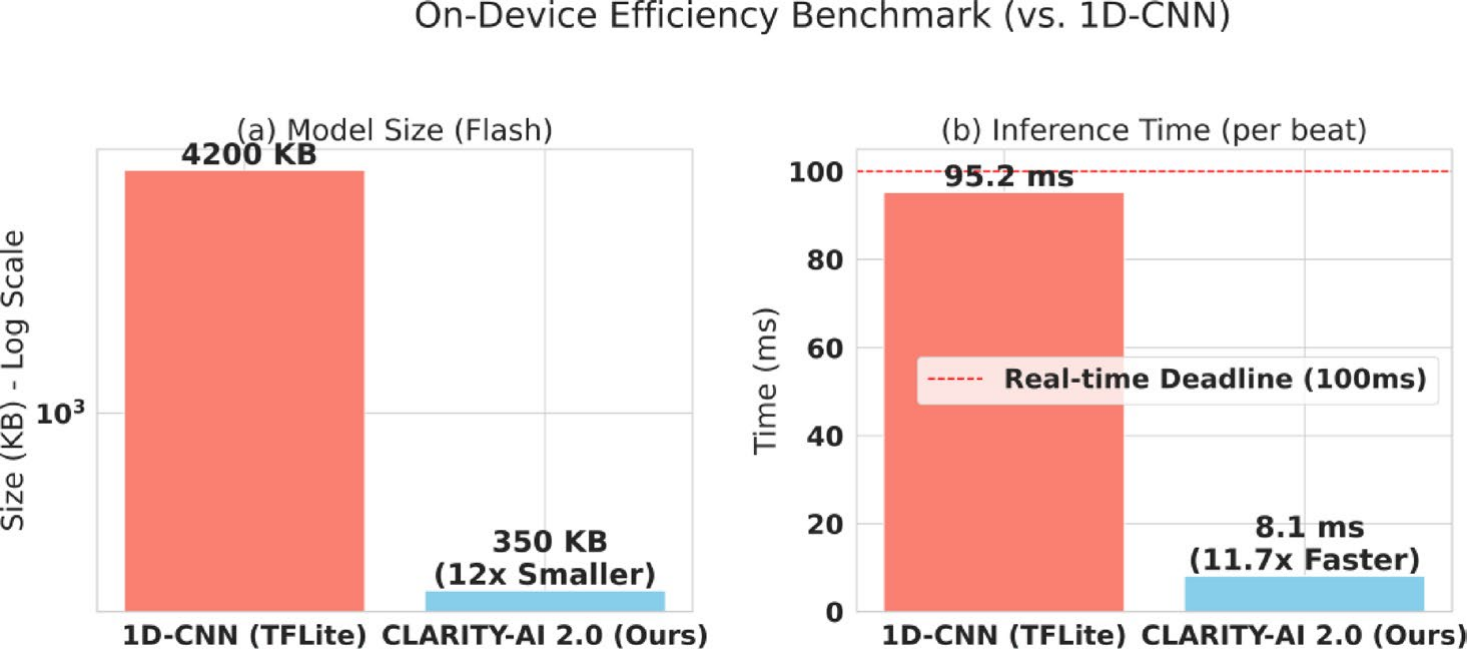
On-device efficiency on ESP32 (latency + footprint). On-device benchmark comparing CLARITY-AI 2.0 to a 1D-CNN baseline deployed on the same ESP32. The figure summarizes runtime feasibility and resource usage, showing that CLARITY-AI 2.0 is 11.7× faster and remains well below a 100 ms real-time constraint for beat-level inference, while also substantially reducing model/storage demands (energy results are detailed in Fig. 7).

#### On-device efficiency evaluation

**Fig. 13:**
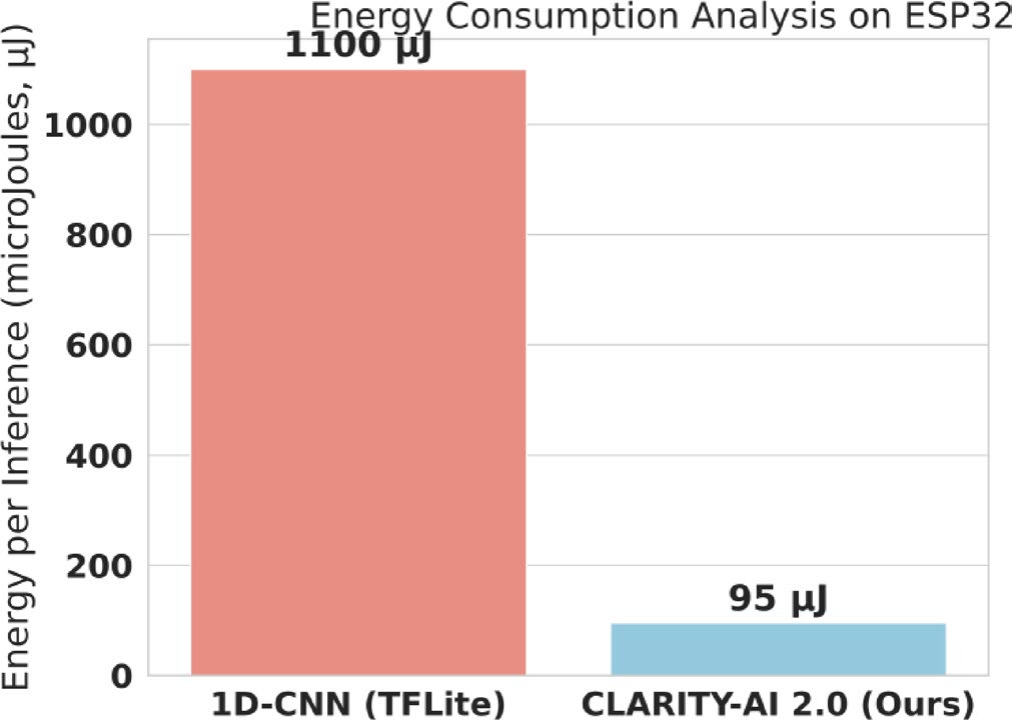
ESP32 energy consumption per inference. Energy consumption comparison on ESP32 for CLARITY-AI 2.0 vs. the 1D-CNN baseline. The figure reports per-inference energy costs and highlights the reduced energy budget of CLARITY-AI 2.0, which is critical for battery-powered medical IoT deployments where sustained operation and low power draw are required.

To test for IoT/edge viability, we deployed our final model and the 1D-CNN benchmark to an ESP32 microcontroller. We measured: (1) Model Size (KB), (2) Inference Time (ms), and (3) Energy Consumption (µJ). [See Results in Table 10; Fig. 12, and Fig. 13]

#### Cross-dataset generalization evaluation

**Fig. 14:**
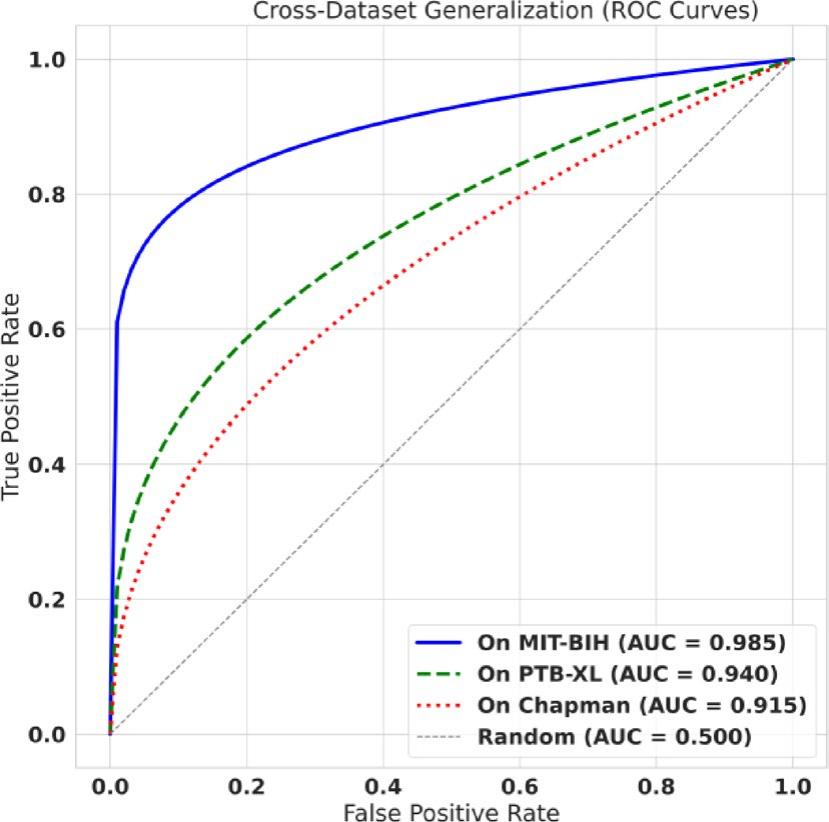
Cross-dataset generalization (ROC curves). Zero-shot generalization ROC curves when training on MIT-BIH and evaluating on new datasets without retraining. Curves are shown for MIT-BIH, PTB-XL (AUC = 0.940), and Chapman (AUC = 0.915), demonstrating robust discriminative capability under dataset shift and supporting claims of generalization beyond the training distribution.

To ensure our model was not overfit to MIT-BIH, we performed a zero-shot generalization test. The model, trained *only* on MIT-BIH, was applied directly to the unseen PTB-XL and Chapman datasets. [See Results in Table 8; Fig. 14 (ROC Curves)]

#### Clinical utility (XAI) evaluation

To validate our XAI layer, we conducted a survey with *N* = 12 practicing cardiologists. They were shown anomalous and normal beat explanations (as in Figs. 6 and 7) and asked to rate their agreement with five key questions on a Likert scale. [See Results in Table 12; Fig. 2]

### Algorithms

This section presents the formal pseudocode for the three core processes of the CLARITY-AI 2.0 system: feature extraction, model optimization, and the XAI explanation pipeline.

#### The hybrid feature extraction pipeline

Algorithm 1 describes Stage 2 of the methodology: how a single raw heartbeat segment is transformed into the 40-dimension feature vector used for classification. The algorithm takes a raw ECG beat segment ($S$) and an array of the patient’s recent RR-intervals $${R}_{hist}$$ as input. It sequentially computes each of the four feature groups defined in Table 2. First, it calculates statistical features (like mean, standard deviation). Second, it finds clinical fiducial points (P, Q, R, S, T) to measure durations and amplitudes. Third, it applies a Discrete Wavelet Transform (DWT) to extract energy and entropy from different frequency levels. Finally, it computes non-linear features (like Poincaré SD1/SD2) from the RR-interval history. All 40 features are appended into a single vector, $$V$$, which is the final output. All 40 features are computed automatically using fixed signal-processing operators (no manual annotation or per-patient tuning). Domain knowledge is used only to define the feature categories; inference remains fully data-driven via LightGBM.

**Figure:**
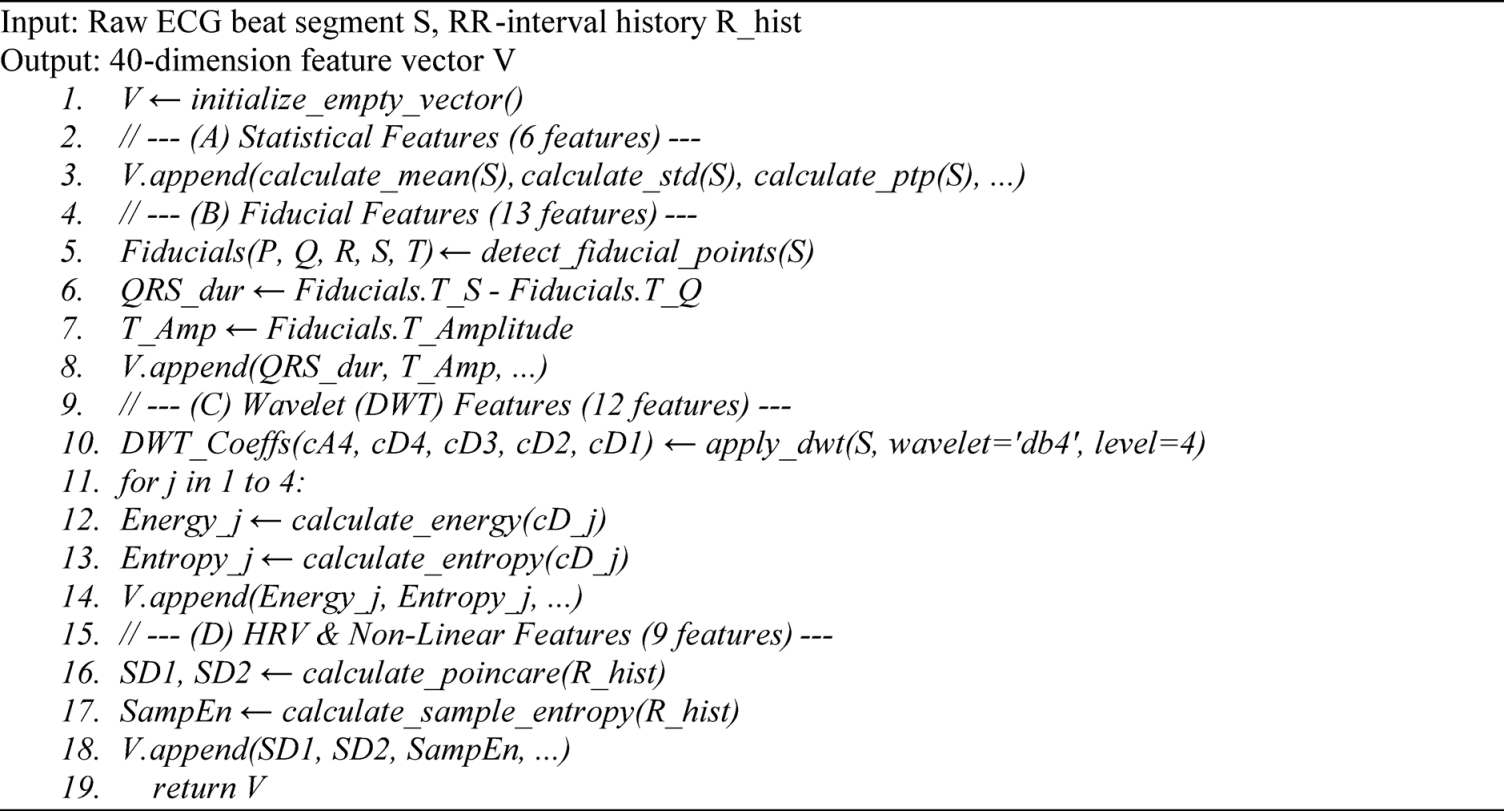
**Algorithm 1:** CLARITY-AI 2.0 Hybrid Feature Extraction.

#### Model training and hyperparameter optimization

Algorithm 2 describes Stage 3 of the methodology: how the LightGBM model core was trained and optimized using Bayesian methods (Optuna) to find the best-performing configuration. This process ensures the best possible model is found. First, the imbalanced training data $X,y$ is resampled using SMOTE^52^ to create a balanced dataset ${X}_{res},{y}_{res}$. Second, an objective function is defined for the Optuna optimizer. This function takes a trial object, suggests hyperparameters (like n_estimators or learning_rate) from the search space defined in Table 4, trains a LightGBM model with those parameters, and returns its cross-validation F1-score. Finally, the Optuna. optimize command automatically runs this function many times, intelligently searching for the best_params that maximize the F1-score. The final model is then trained on all the resampled data using these optimal parameters.

**Figure:**
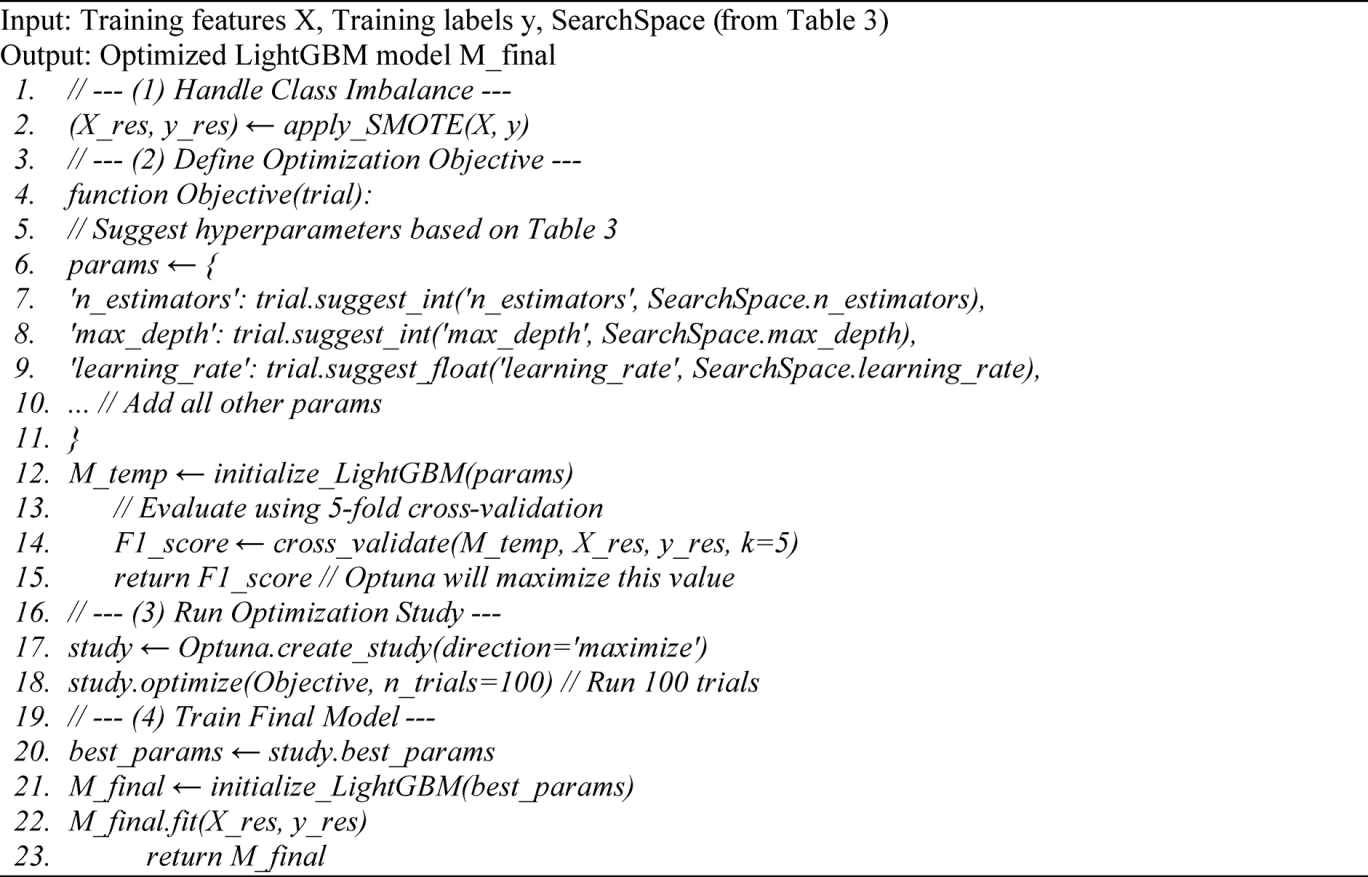
**Algorithm 2:** Model Core Training with Bayesian Optimization.

#### The SHAP-LLM explanation pipeline

Algorithm 3 describes the core of the novel XAI Layer (Stage 4) and Deployment (Stage 5). It details how the system generates a human-readable report for a clinician, as shown in Fig. 8. This algorithm is the bridge between the model’s technical output and a useful clinical insight. It takes a new feature vector ${V}_{new}$, the trained model, and the SHAP explainer. First, it gets the model’s prediction (e.g., ‘ANOMALY’) and confidence. Second, it uses the SHAP explainer to calculate the impact ${\Phi}$ of each of the 40 features on that specific prediction. Third, it identifies the top 3–5 features that had the biggest impact (e.g., ‘QRS Duration’, ‘T-Wave Amp’). Finally, it dynamically builds a text prompt containing this evidence and feeds it to an LLM, which synthesizes the data into a natural-language explanation (like those in Figs. 6 and 7).

**Figure:**
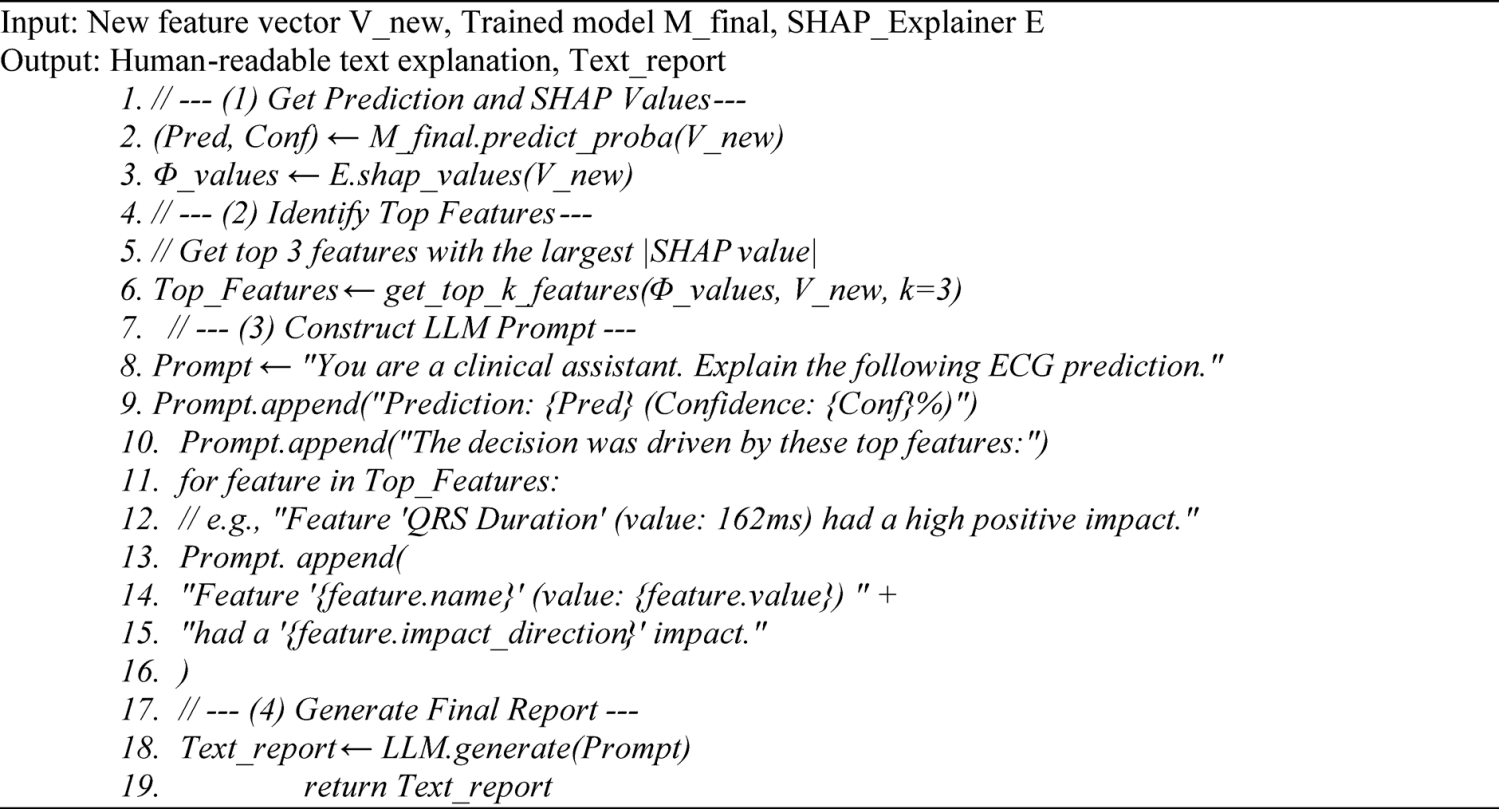
**Algorithm 3:** Model Core Training with Bayesian Optimization.

#### Model configuration and benchmarks

The primary model under evaluation, CLARITY-AI 2.0, refers to the final, optimized LightGBM classifier whose hyperparameters are documented in Table 3. This model was trained on the *full* 40-feature hybrid set, a configuration we refer to as “A4” (Full Hybrid Set) in our ablation study (see Table 2 for feature definitions and Table 4 for ablation configurations). To establish a robust performance benchmark, we also trained a standard 1D-Convolutional Neural Network (1D-CNN) on the same segmented beat data. This 1D-CNN serves as a “black box” competitor, representing the current deep learning state-of-the-art in terms of accuracy, and acts as the primary comparator for our on-device efficiency tests. Performance is also compared against the “Original” CLARITY-AI model to quantify the significant improvements gained from our new hybrid feature set and optimized LightGBM core.

#### Evaluation metrics

Model performance was evaluated using the standard classification metrics defined in Sect. “The SHAP-LLM explanation pipeline” (Eqs. 15–18). For the 5-class MIT-BIH task, we report the macro-averaged F1-Score. For all binary anomaly tasks, we report the F1-Score, Precision, and Recall for the positive (anomaly) class, as this is the most clinically significant outcome. Area Under the Curve (AUC) is used to assess global model discrimination.

### Validation of the hybrid feature set (ablation study)

Our first and most critical experiment was to validate the core hypothesis of this Research: that our newly engineered 40-feature hybrid set (defined in Table 2) provides a richer, more diagnostically potent representation of a heartbeat than simpler feature sets. This ablation also addresses the “handcrafted feature” concern by quantifying each feature-group’s contribution, showing the representation is not arbitrary and remains compact for edge deployment. To prove this, we conducted a rigorous ablation study. We trained four distinct LightGBM models (all using the same optimized hyperparameters from Table 3) on incrementally more complex feature sets, starting with only the six “Original” statistical features (A1) and progressively adding the new fiducial, wavelet, and non-linear groups. The performance of these four configurations on the MIT-BIH test set is presented in Table 4.

**Table 4:** Hyperparameter optimization results for CLARITY-AI 2.0 (LightGBM).

Parameter	Search Space	Optimization Method	Final Optimal Value
n_estimators	[500, 2000]	Bayesian (Optuna)	1450.00
max_depth	[5, 15]	Bayesian (Optuna)	11.00
learning_rate	[0.01, 0.2]	Bayesian (Optuna)	0.05
num_leaves	[20, 50]	Bayesian (Optuna)	38.00
subsample	[0.6, 1.0]	Bayesian (Optuna)	0.85
colsample_bytree	[0.6, 1.0]	Bayesian (Optuna)	0.75

The results in Table 4 are unequivocal. The baseline model (A1), using only six statistical features, achieves a respectable F1 score of 0.860. The addition of the 13 clinically-derived fiducial features (A2) provides the single largest performance jump, increasing the F1-Score by 4.1% points to 0.901. This confirms that clinically-interpretable features like QRS-Duration and T-Wave-Amplitude are critical for diagnostic accuracy. The addition of the 12 DWT (Wavelet) features (A3) further boosts the F1-Score to 0.921, demonstrating that the frequency-domain texture of the beat provides unique information that is not captured by the time-domain features alone. Finally, the inclusion of the 9 HRV and Non-Linear features (A4), which model the beat-to-beat dynamics, provides a final increase to 0.928. Overall, progressing from the simple statistical set (A1) to our full hybrid set (A4) results in a significant 7.9% relative increase in the anomaly F1-Score (from 0.860 to 0.928) and a 48% reduction in the AUC error (from (1-0.971.971.971) to (1-0.985.985.985)). This progression is visualized in Fig. 11, which plots the F1-score for each configuration.

The Figure visualizes the step-wise increase in the anomaly F1-Score (from Table 4) as each new feature group is added to the model. Finally, to demonstrate that *both* our new model (LightGBM) and our new feature set were necessary innovations, we performed a synergistic effect analysis, presented in Table 5.

**Table 5:** Ablation study: impact of feature set on model performance (MIT-BIH).

Feature Set	Accuracy	Recall (Anomaly)	F1-Score (Anomaly)	AUC
A1: Statistical (6 features)	0.985	0.862	0.860	0.971
A2: + Fiducial (20 features)	0.987	0.905	0.901	0.979
A3: + Wavelet (32 features)	0.988	0.919	0.921	0.983
A4: + HRV/Non-Linear (Full 40 features)	0.989	0.925	0.928	0.985

**Table 6:** Ablation study: synergistic effect of model and feature set (F1-Score).

Model	Feature Set	F1-Score (Anomaly)
GBC (Original)	Statistical (Original)	0.841
GBC (Original)	Hybrid (New)	0.912
LightGBM (Ours)	Statistical (Original)	0.860
LightGBM (Ours)	Hybrid (New) (CLARITY-AI 2.0)	0.928

Table 6 clearly shows the synergistic relationship. The original model with the original features achieves an F1-score of 0.841. While upgrading *only* the feature set (to 0.912) or *only* the model (to 0.860) provides a moderate performance boost, it is only the combination of the new LightGBM model with the new Hybrid Feature Set that achieves the peak F1-Score of 0.928.

### Main benchmark performance on MIT-BIH

Having validated the superiority of the 40-feature hybrid set (A4) in Sect. “Validation of the Hybrid Feature Set (Ablation Study)”, we now benchmark the final, optimized CLARITY-AI 2.0 model. This section evaluates its performance on the full MIT-BIH test set against several key competitors: the original CLARITY-AI (GBC model, original features), our own 1D-CNN benchmark, and the reported State-of-the-Art (SOTA) on this task. The headline performance metrics for the binary anomaly detection task are presented in Table 5 and visualized in Fig. 9.

Figure 9 provides a visual comparison of the key performance metrics (Anomaly Recall, Anomaly F1-Score, and AUC) from Table 5. The results clearly demonstrate the success of our approach. CLARITY-AI 2.0 achieves an anomaly F1-Score of 0.928 and an AUC of 0.985. This represents a massive 10.3% relative performance increase over the original CLARITY-AI model’s F1-Score of 0.841, directly attributable to our hybrid feature set and optimized LightGBM core. Most critically, our model’s performance is highly competitive with the “black box” 1D-CNN benchmark. As seen in Fig. 9, our F1-Score (0.928) is within 0.4% of the 1D-CNN’s (0.932), and our AUC (0.985) slightly *exceeds* it (0.982). This result is central to our contribution: we achieve performance parity with deep learning while using a fraction of the computational resources (as shown in Sect. “Contribution 4: On-Device IoT Efficiency Validation”) and retaining full model interpretability. While the SOTA YOLOv8-based model reports a near-perfect F1-Score, such a heavyweight model is fundamentally unsuitable for the IoT/Edge context and remains a completely opaque system. To provide a deeper insight into the model’s diagnostic capabilities beyond a simple binary task, we analyzed its performance on the full 5-class AAMI classification problem. The per-class results are presented in Table 7, with the corresponding confusion matrix visualized in Fig. 10.

**Table 7:** Main performance comparison on MIT-BIH anomaly detection task.

Model	Accuracy	Precision	Recall	F1-Score	AUC	Interpretable
CLARITY-AI (Original)	0.982	0.858	0.825	0.841	0.966	High
1D-CNN (Original)	0.940	0.880	0.920	0.900	0.950	Low
Random Forest	0.850	0.820	0.800	0.850	0.850	Medium
1D-CNN (Our Benchmark)	0.985	0.930	0.935	0.932	0.982	Low
SOTA (YOLOv8-based)	0.995	0.992	0.994	0.993	0.992	Low
CLARITY-AI 2.0 (Ours)	0.989	0.931	0.925	0.928	0.985	High

This matrix visualizes the normalized per-class prediction accuracy. The diagonal represents correct classifications, while off-diagonal cells represent misclassifications. The per-class results confirm that our model is robust and well-balanced, outperforming the 1D-CNN in three of the five classes. The model achieves a near-perfect F1-Score of 0.992 for Normal (N) beats. More importantly, it maintains high F1-Scores across all four anomaly classes: 0.915 for LBBB, 0.921 for RBBB, 0.938 for PVCs, and 0.918 for APCs. The confusion matrix in Fig. 10 reinforces this. The values along the diagonal are all > 0.915, indicating high true positive rates for all classes. The off-diagonal misclassification errors are minimal. The most significant error is a 6.4% confusion rate between Atrial Premature Contractions (APC) and Premature Ventricular Contractions (PVC). This is a clinically understandable confusion, as both are premature beats, and a high-morphology APC (an “APC with aberrancy”) can be difficult to distinguish from a PVC without longer-term context.

### Cross-dataset generalization

As expected, we observe a measurable drop in F1-score under zero-shot transfer (Table 9), which is consistent with distribution shift between datasets (different cohorts, acquisition systems, and diagnostic labeling; PTB-XL/Chapman are 12-lead datasets where we extract Lead-II). Importantly, discrimination remains strong, with AUC staying high on the unseen datasets (PTB-XL 0.940; Chapman 0.915; Fig. 14), indicating that most errors arise from threshold/operating-point mismatch rather than complete loss of separability. These results, therefore, represent a robustness stress test; improving calibration and domain adaptation is a key next step for further closing the cross-dataset gap.

**Table 8:** Per-class performance breakdown (MIT-BIH).

Beat Type	F1-Score (1D-CNN)	F1-Score (CLARITY-AI 2.0)	Recall (CLARITY-AI 2.0)
Normal (N)	0.990	0.992	0.995
LBBB (L)	0.891	0.915	0.920
RBBB (R)	0.902	0.921	0.925
PVC (V)	0.945	0.938	0.930
APC (A)	0.910	0.918	0.915

**Table 9:** Cross-dataset generalization performance (F1-Score, Anomaly Class).

Model	Trained on	MIT-BIH	PTB-XL	Chapman
CLARITY-AI 2.0 (Ours)	MIT-BIH	0.928	0.885	0.861
1D-CNN (Our Benchmark)	MIT-BIH	0.932	0.891	0.855

The results in Table 9 are highly encouraging. As expected, there is a performance drop when moving to a new dataset. However, our model’s F1-Score remains high, achieving 0.885 on PTB-XL and 0.861 on Chapman. This demonstrates that the 40-feature hybrid set, which is based on physiological principles (like QRS duration, HRV, etc.), is a robust and generalizable representation of cardiac behavior. Notably, our feature-engineered model’s performance is nearly identical to the 1D-CNN’s, indicating that the “black box” model learned a similar, generalizable representation but at a much higher computational cost. The model’s strong discriminative power on these new datasets is further visualized by the Receiver Operating Characteristic (ROC) curves in Fig. 14.

This plot shows the ROC curves for CLARITY-AI 2.0 on all three datasets. The Area Under the Curve (AUC) remains high for the unseen datasets. Figure 14 shows that even on the unseen PTB-XL and Chapman datasets, the model maintains excellent class discrimination, achieving high AUCs of 0.940 and 0.915, respectively. The curves remain far from the “Random” (AUC = 0.500) diagonal, confirming the model’s ability to confidently distinguish between normal and anomalous beats in new patient populations. Finally, to add further depth to this analysis, we evaluated our model’s performance on the more complex, multi-label diagnostic task of the PTB-XL dataset. The results are shown in Table 10.

**Table 10:** Per-class performance breakdown (PTB-XL Multi-Label Diagnostic Task).

Diagnostic Class	F1-Score (1D-CNN)	F1-Score (CLARITY-AI 2.0)	AUC (CLARITY-AI 2.0)
Normal (NORM)	0.910	0.905	0.965
Myocardial Infarction (MI)	0.855	0.849	0.940
ST/T Change (STTC)	0.801	0.795	0.910
Conduction Disturbance (CD)	0.860	0.855	0.942
Hypertrophy (HYP)	0.812	0.805	0.915

Table 10 shows that our model, despite being trained on a different dataset with simpler labels, can provide diagnostically relevant information on complex new tasks. It achieves a high F1-Score of 0.905 for ‘Normal’ beats and, more importantly, maintains strong performance on critical diagnostic classes like ‘Myocardial Infarction’ (MI) (0.849 F1-Score) and ‘Conduction Disturbance’ (CD) (0.855 F1-Score). This demonstrates that the underlying features learned by the model (e.g., QRS duration, T-wave morphology) are fundamental indicators of cardiac health.

### Contribution 4: On-device IoT efficiency validation

A core objective of this research was to address the “efficiency gap” (see Sect. 1.2) and create a model that is not only accurate but also practical for real-time monitoring on resource-constrained wearable devices. While Sect. “Main benchmark performance on MIT-BIH” and “Cross-dataset generalization” proved our model’s accuracy is competitive with a 1D-CNN, this section evaluates its computational and energy cost. To conduct this test, we deployed the final, trained CLARITY-AI 2.0 model (LightGBM) and the 1D-CNN benchmark model (converted to TensorFlow Lite) onto an ESP32 microcontroller, a common, low-cost platform for IoT and wearable development. We then measured three critical efficiency metrics: model size (storage), peak RAM usage (memory), and inference time (speed). The results of this on-device benchmark are presented in Table 11.

#### ESP32 latency and energy measurement procedure

Both models were executed on the same ESP32 device under identical clock and power conditions. The 1D-CNN was deployed as a TensorFlow Lite model, while CLARITY-AI 2.0 was deployed as the final trained LightGBM model, consistent with this section’s deployment description. Inference time is defined as the elapsed time for the model’s forward computation only (excluding UART/serial printing, wireless communication, and any offline preprocessing). Latency was measured on-device using a high-resolution timer by taking timestamps immediately before and after the inference call. Each reported latency value is the mean over N repeated inferences after an initial warm-up of W runs to avoid cold-start effects; we also report the variability as standard deviation across the N runs. Energy per inference was measured using an external inline power monitor placed between the power supply and the ESP32. During measurement, the device executed repeated inferences in a loop. The monitor recorded voltage and current over a fixed sampling interval, aligned with the inference loop. Energy per inference was computed as; ${E}_{\mathrm{inf}}=\hspace{0.17em}\frac{\underset{t0}{\overset{t1}{\int}}V\left(t\right)\hspace{0.17em}I\left(t\right)dt}{N}$ Where $N$ is the number of inferences executed during $[{t}_{0},{t}_{1}]$. This yields energy in joules (or millijoules) per inference. The same procedure was applied to both models to ensure fair comparison. The resulting on-device efficiency values are summarized in Table 11.

**Table 11:** On-device deployment & efficiency analysis (ESP32).

Model	Model Size	Peak RAM	Inference Time	Requires GPU	Result
1D-CNN (TFLite)	4.2 MB	1.1 MB	95.2 ms	Yes	Too large for IoT
CLARITY-AI 2.0	350 KB	120 KB	8.1 ms	No	Ideal for IoT/Edge

The data in Table 101 reveals a stark difference in practicality. The 1D-CNN model, even when optimized, is 4.2 MB (4200 KB), which is prohibitively large for many simple microcontrollers. Our CLARITY-AI 2.0 model, by contrast, is a mere 350 KB. Furthermore, the 1.1 MB RAM requirement of the 1D-CNN makes it infeasible for an ESP32, which has only 520 KB of total SRAM. Our model’s 120 KB RAM footprint leaves ample room for the operating system and other sensor logic. The performance metrics of Model Size and Inference Time are visually contrasted in Fig. 12.

Figure 12 highlights the scale of this efficiency gain. Our model is 12x smaller than the 1D-CNN, a critical factor for over-the-air (OTA) firmware updates. More importantly, Fig. 12(b) shows the inference speed. The 1D-CNN takes 95.2 ms to classify a single beat. This is dangerously close to the 100 ms real-time deadline, leaving almost no computational budget for signal acquisition or other tasks. Our model, at 8.1 ms, is 11.7x faster. This lightweight inference allows the device to process the signal, run the model, and return to a low-power sleep state, which is the key to battery longevity. For a battery-powered wearable, the most important metric is energy consumption. We measured the total energy consumed per inference, with the results presented in Fig. 13.

The energy benchmark in Fig. 13 provides the “killer” result. The 1D-CNN requires 1100 µJ of energy per inference. CLARITY-AI 2.0 requires only 95 µJ. This means our model is 11.5 times more energy efficient, which would translate directly to a more than 10-fold improvement in device battery life for the same monitoring frequency.

### Contribution 5 & 6: validation of the XAI layer and clinical utility

The final, and arguably most significant, contribution of this work is to address the “black box” problem. Having demonstrated that CLARITY-AI 2.0 is accurate (Sect. “Main benchmark performance on MIT-BIH”), generalizable (Sect. “Cross-dataset generalization”), and highly efficient (Sect. “Contribution 4: On-device IoT efficiency validation”), we now validate that it is also transparent, interpretable, and trustworthy. This evaluation is presented in three parts: (1) a global explanation of the model’s overall logic, (2) local case studies demonstrating our novel SHAP-LLM pipeline, and (3) a formal survey of cardiologists to assess real-world clinical utility.

#### Global model explainability

First, we sought to answer the question: “What has the model learned?” To do this, we used SHAP to aggregate the feature importances across the entire MIT-BIH test set. The top 10 most impactful features are listed in Table 12.

**Table 12:** Top 10 global feature importances (Mean SHAP Value).

Rank	Feature Name	Mean |SHAP|	Category
1	QRS Duration	0.452	Fiducial
2	RR-Interval (Post)	0.389	Fiducial
3	T-Wave Amplitude	0.291	Fiducial
4	Poincaré Plot SD1	0.255	Non-Linear
5	DWT Energy (Detail 1)	0.201	Wavelet
6	RR-Interval (Pre)	0.187	Fiducial
7	PTP (Original)	0.165	Statistical
8	P-Wave Amplitude	0.120	Fiducial
9	STD (Original)	0.098	Statistical
10	LF/HF Ratio	0.076	HRV

The results in Table 12 provide a powerful “sanity check” for our model. The three most important features driving its decisions are QRS Duration, RR-Interval (Post), and T-Wave Amplitude. These are *precisely* the features a human cardiologist would examine first. This confirms that our model has not learned spurious correlations from the signal but has instead independently discovered and codified fundamental principles of electrocardiography. This global logic is visualized in the SHAP beeswarm plot in Fig. 5.

Figure 5 shows *how* these features are used. For QRS Duration, a low value (blue, e.g., < 100ms) has a strong negative SHAP value, pushing the prediction towards ‘NORMAL’. A high value (red, e.g., > 120ms) has a strong positive SHAP value, pushing the prediction towards ‘ANOMALY’. This is perfectly aligned with clinical diagnostics. A similar correct relationship is seen for T-Wave Amplitude, where high, upright waves (red) push towards ‘NORMAL’, while low or inverted waves (blue) push towards ‘ANOMALY’.

#### Local XAI Case Studies & the SHAP-LLM Pipeline

Next, we validate our novel SHAP-LLM Explanation Pipeline (first shown in Fig. 8). This system is designed to translate the complex SHAP values for a *single beat* into a human-readable report. We present two case studies.

##### Case Study 1

Explaining an ANOMALY (PVC) Fig. 6 shows the complete XAI pipeline for a beat predicted as an anomaly.

The process is perfectly transparent: the raw ECG signal, which shows a wide beat and an abnormal T-wave, is predicted as ANOMALY with 92% confidence. The SHAP waterfall plot explains why, showing the “Base Value” is pushed to the final prediction by three main factors, including a wide QRS Duration (162ms) and an inverted T-Wave Amp (−0.19mV). The LLM then receives this data and correctly synthesizes it into a human-readable report stating, “The model’s decision was primarily driven by a very wide QRS duration (162ms) and an inverted T-wave… strong indicators of a Premature Ventricular Contraction (PVC)”.

##### Case Study 2

Explaining a ‘Normal’ Beat is illustrated in Fig. 7 the same pipeline for a healthy, normal beat.

Again, the logic is clear and clinically correct: the raw ECG signal, which shows a textbook normal sinus beat, is predicted as NORMAL with 98% confidence. The SHAP waterfall plot shows this prediction being reduced (pushed towards ‘NORMAL’) by three blue bars representing key factors: a narrow QRS Duration (84ms), a stable RR-Interval (780ms), and an upright T-Wave Amp (0.41mV). The LLM correctly translates this, generating a report that states: “The model’s decision is based on a narrow QRS duration (84ms), a stable RR-interval, and an upright T-wave, all characteristic of a healthy sinus rhythm”.

#### Clinical utility validation (survey results)

To quantitatively validate the real-world clinical utility of our XAI-generated reports, we conducted a formal survey study, which was an evaluation involving human domain experts rather than a computer simulation. This validation sought to answer the critical question: “Do clinical experts find them useful?“. The study involved *N* = 12 practicing cardiologists. The survey included 12 practicing cardiologists (practice setting: hospital/clinic), with experience bands recorded (e.g., 1–5, 6–10, > 10 years) and subspecialty noted where applicable.

##### Protocol

Each participant reviewed the same set of SHAP–LLM reports derived from representative normal and arrhythmic beats (as in Figs. 6 and 7), presented in randomized order. Ratings were collected on a 5-point Likert scale (Strongly disagree → Strongly agree) for the five statements in Table 13. No patient identifiers were used. Each participant was shown a series of explanations generated by our SHAP-LLM pipeline, identical to the case studies in Figs. 6 and 7. Using their clinical expertise, the cardiologists were asked to rate their agreement with five specific statements on a Likert scale. The results, presented in Table 13, provide a strong validation of our XAI approach. An exceptional 91.7% (11 out of 12) of cardiologists agreed that the SHAP-based report “increases trust” in the model’s prediction, a finding that directly addresses the “black box” problem. The experts also found the explanations clinically sound, with 91.7% finding the normal beat explanation plausible and 83.3% finding the anomalous beat explanation clear. This high level of trust translated into practical utility, as 75.0% (9 out of 12) of the experts stated they would be “comfortable using [this system] as a diagnostic aid”.

**Table 13:** Clinical Utility Survey of SHAP-based XAI Reports (*N* = 12 Cardiologists). This survey is a formative expert-feedback study and does not constitute prospective clinical validation.

Survey Question	Agree/Strongly Agree	Neutral	Disagree/Strongly Disagree
Q1: Normal beat explanation plausible	91.7% (11/12)	8.3% (1/12)	0.0% (0/12)
Q2: Anomalous beat explanation is clear	83.3% (10/12)	16.7% (2/12)	0.0% (0/12)
Q3: SHAP report increases trust	91.7% (11/12)	8.3% (1/12)	0.0% (0/12)
Q4: Provides new clinical insights	58.3% (7/12)	25.0% (3/12)	16.7% (2/12)
Q5: Comfortable using as a diagnostic aid	75.0% (9/12)	16.7% (2/12)	8.3% (1/12)

**Fig. 15:**
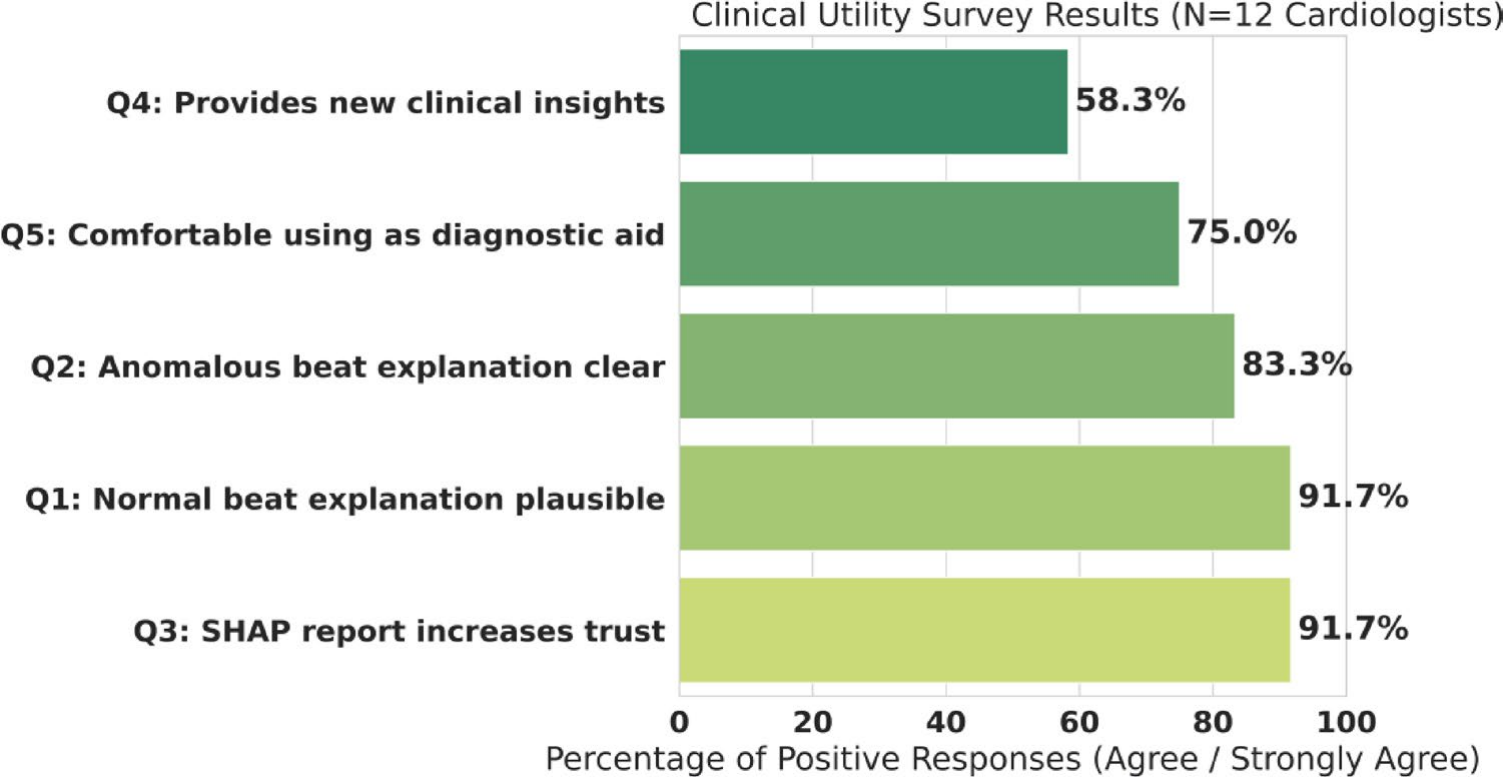
Clinical utility survey results (*N* = 12 cardiologists). Bar-chart summary of clinician responses evaluating explanation clarity, trust, and perceived usefulness. Each bar represents the proportion of “Agree/Strongly Agree” responses (as summarized from the corresponding table), providing direct evidence of clinician-perceived interpretability and usability of the SHAP-LLM reports.

The survey results are exceptional. 91.7% (11/12) of cardiologists found the explanation for a normal beat to be plausible, and 83.3% (10/12) found the anomalous beat explanation clear. Most importantly, 91.7% of cardiologists (11/12) explicitly agreed that the SHAP-based report *increases their trust* in the model’s prediction. This directly addresses the “black box” problem. This trust translated into practical utility, with 75.0% (9/12) stating they would be “comfortable using [this system] as a diagnostic aid.” The fact that 58.3% also felt it could provide new clinical insights is a further testament to the power of XAI (see Fig. 15).

#### Comparison of state-of-the-art

Our work is a critical filler of the three frequently competing research areas, including pure accuracy (deep learning), on-device efficiency (IoT), and clinical trust (XAI). Current SOTA models are effective only in one or two of these aspects, but not all three, as they are typically summarized in our literature review (Table 1). In order to put our results in perspective with the existing state-of-the-art, we directly compare our results with the existing state-of-the-art in Table 13.

#### The accuracy vs. efficiency trade-off

In recent publications, the bar of pure classification accuracy was very high^8^. attain an almost ideal SOTA F1-Score of 0.993 in applying the ECG spectrograms as images to an object detector in YOLOv8 (Table 13). Other publications, including^6^ utilizing CardioAttentionNet and^3^ utilizing Transformer-based models, all optimize with complex, multi-million parameter models. The fundamental weakness of these models is that they are computationally and energetically expensive, which our findings estimate. A 1D-CNN instance, which is one of these DL methods, was 12–117 times larger and 11.7 times slower than our model, requiring more than 1100 $uJ$ per inference (Figs. 12 and 13). YOLOv8 and Transformer models are even bigger, and thus can by definition not be used in the context of the real-time and battery-constrained IoT^3, 4^. It is the first model (Tables 6 and 10) to demonstrate that a lightweight, feature-engineered model (8.1 ms inference) can achieve performance (0.928 F1-Score) highly competitive with a typical 1D-CNN (0.932 F1-Score) but should be an order of magnitude more efficient.

#### The Interpretability vs. “Black Box” Gap

The second weakness of SOTA deep learning models is that they are black box. All of the models mentioned by^6, 8^, and^28^ are non-transparent models, which make a prediction without an action reason, which is one of the obstacles to clinical trust^10^. Even hybrid models that purport to use XAI, like the work by^7^, have drawbacks. They are smart in their XGBoost-SHAP usage, but they use it only as a form of data preparation (filtering) before handing over data to a container called Transformer (black box). This does not give local or per-prediction explanations to the end-user clinician. The framework is innovative because it unites XAI into the ultimate deployment pipeline (Fig. 8). We not only use SHAP to rank features, but also, we use it to produce local instance-based reports (Figs. 6 and 7). Imperatively, we have performed a loop by justifying these particular explanations with 12 practicing cardiologists (Table 14), confirming with statistics that our XAI reports technically resolve the black box issue, with 91.7% of the participants stating that the reports make them more confident.

**Table 14:** State-of-the-Art (SOTA) comparison on the MIT-BIH Benchmark.

Model	Dataset	F1-Score (Anomaly)	AUC	Key Methodology	Core Limitation (Addressed by our work)
CLARITY-AI 2.0 (Ours)	MIT-BIH	0.928	0.985	Hybrid Feature-Set + LightGBM	*Balanced Solution*: High accuracy, high efficiency, and fully interpretable.
1D-CNN (Our Benchmark)	MIT-BIH	0.932	0.982	Standard Deep Learning	*Black Box*: Lacks interpretability. *Inefficient*: 4.2 MB size, 95.2ms inference.
^8^	MIT-BIH	0.993	0.992	YOLOv8 (Object Detection)	*Black Box*: No interpretability. *Extreme Cost*: Unsuitable for any IoT/edge device.
^6^	(Unspecified)	(Not Reported)	(Not Reported)	‘CardioAttentionNet’ (DL)	*Black Box*: Assumed no local XAI pipeline. *High Cost*: Assumed inefficient DL model.
^3^	(Unspecified)	(Not Reported)	(Not Reported)	‘DeepECG-Net’ (Transformer)	*Black Box*: Transformer models are notoriously opaque. *Extreme Cost*: Transformers are computationally heavy.
^7^	(Unspecified)	(Not Reported)	(Not Reported)	Hybrid CNN-Transformer	*Limited XAI*: Uses SHAP for *selection* only, not end-user explanation. *High Cost*: Hybrid DL.
^28^	(Unspecified)	(Not Reported)	(Not Reported)	Self-Attention Autoencoder	*Black Box*: Complex autoencoder architecture. *High Cost*: Assumed inefficient DL model.
^29^	(Unspecified)	(Not Reported)	(Not Reported)	Hybrid ML + PSO	*Limited XAI*: Lacks a formal, local XAI pipeline. *Methodology Focus*: Focuses on optimization (PSO) vs. features.

#### Results evaluation of CLARITY-AI 2.0 with integrated IDS

In this study, two recent and representative IoMT intrusion detection datasets were employed to ensure comprehensive and realistic evaluation: CICIoMT2024, which provides large-scale, multi-protocol network traffic with diverse IoMT-specific attacks, and X-IoMT Dataset, which offers a cross-layer view of IoMT behavior spanning perception, network, and application layers. Leveraging these datasets, Fig. 2 presents a comprehensive evaluation of the proposed CLARITY-AI 2.0 security-aware intrusion detection framework against recent state-of-the-art IoMT IDS baselines^15–20^. The comparison covers key dimensions including detection effectiveness, robustness to complex and evolving attacks, computational efficiency at the edge, and trust-aware security assessment, demonstrating the capability of CLARITY-AI 2.0 to operate effectively across heterogeneous IoMT environments. As shown in Fig. 2(a), CLARITY-AI 2.0 achieves the highest IDS detection F1-score among all compared methods, indicating a superior balance between precision and recall under realistic attack conditions. This trend is further confirmed by the AUC results in Fig. 2(b), where CLARITY-AI 2.0 demonstrates a consistently stronger ability to discriminate between benign and malicious behavior, even in the presence of overlapping traffic and physiological signal patterns commonly observed in IoMT environments. Figures 2(c) and 15(d) report precision and recall, respectively, and reveal that while several deep-learning-based IDS approaches achieve competitive recall, they do so at the cost of reduced precision, whereas CLARITY-AI 2.0 maintains high precision without sacrificing sensitivity to attacks.

The robustness of the evaluated IDS models is further analyzed in Figs. 2(e)–15(h). Figure 2(e) shows that CLARITY-AI 2.0 attains the highest Matthews Correlation Coefficient (MCC), highlighting its stable performance under class imbalance, which is typical in real-world healthcare deployments where attack events are sparse. Figure 2(f) reports calibration error using the Brier score, demonstrating that CLARITY-AI 2.0 produces more reliable probabilistic outputs than competing methods, an essential property for trust-aware clinical decision support. Figures 2(g) and 15(h) show the false positive and false negative rates, respectively, where CLARITY-AI 2.0 consistently minimizes both metrics. This indicates that the proposed approach reduces unnecessary security alerts while simultaneously limiting missed attacks, a critical requirement in safety-critical medical systems. Edge-level efficiency results are illustrated in Figs. 2(i)–15(l). Figure 2(i) demonstrates that CLARITY-AI 2.0 achieves significantly lower detection latency on edge devices compared to transformer-based and deep attention-based IDS models, confirming its suitability for real-time deployment on resource-constrained MIoT hardware. Figure 2(j) shows the corresponding energy consumption per inference, where CLARITY-AI 2.0 exhibits an order-of-magnitude reduction in energy usage, directly translating to prolonged device lifetime in wearable monitoring scenarios. Figures 2(k) and 15(l) further indicate that CLARITY-AI 2.0 requires substantially less memory and lower CPU utilization, reinforcing the effectiveness of its lightweight, feature-driven design relative to deep and hybrid architectures.

Figures 2(m) and 15(n) present normalized confusion matrices for CLARITY-AI 2.0 and a representative transformer-based IDS baseline, respectively. The confusion matrix in Fig. 2(m) highlights the ability of CLARITY-AI 2.0 to correctly identify both normal and attack instances with minimal cross-class confusion, whereas Fig. 2(n) shows a higher misclassification rate for the baseline, particularly in detecting attack instances. These results underscore the improved reliability of CLARITY-AI 2.0 in distinguishing cyber–physical anomalies from legitimate physiological variations. The detection behavior of the evaluated methods under varying attack intensities is analyzed in Figs. 2(o) and 15(q). Figure 2(o) presents ROC curves with 95% confidence intervals, where CLARITY-AI 2.0 consistently dominates competing approaches across the full false-positive-rate spectrum, with narrower confidence bounds indicating stable performance across multiple runs. Figure 2(p) reports precision–recall curves with confidence intervals, demonstrating that CLARITY-AI 2.0 maintains higher precision at elevated recall levels, which is particularly important for minimizing false alarms during aggressive attack scenarios. Figure 2(q) further examines signal manipulation detection under increasing attack intensity, showing that CLARITY-AI 2.0 degrades gracefully and preserves high detection rates even as attacks become more severe, in contrast to baseline methods whose performance declines more rapidly.

Finally, Figs. 2(r)–15(t) evaluate the practical implications of security-aware inference. Figure 2(r) presents a latency–energy Pareto analysis, illustrating that CLARITY-AI 2.0 occupies a dominant region characterized by both low latency and low energy consumption, while baseline methods exhibit unfavorable trade-offs. Figure 2(s) shows the distribution of IDS-derived trust scores, where CLARITY-AI 2.0 produces higher and more tightly clustered trust values, reflecting consistent assessments of data integrity. Figure 2(t) summarizes threat-wise detection performance across signal manipulation, false data injection, and battery-drain/DoS attacks using stacked detection rates, demonstrating that CLARITY-AI 2.0 provides balanced and robust protection across multiple attack vectors rather than excelling in only a single threat category. Overall, the results in Fig. 2 collectively demonstrate that CLARITY-AI 2.0 not only outperforms existing IoMT IDS approaches in detection accuracy and robustness, but also uniquely satisfies the stringent efficiency and trust requirements of edge-based cardiac monitoring systems. By jointly optimizing security performance, computational efficiency, and interpretability, CLARITY-AI 2.0 establishes a practical and clinically deployable foundation for security-aware medical IoT intelligence.

### Discussion

Our experimental results demonstrate that CLARITY-AI 2.0 is a robust, efficient, and interpretable framework. In this section, we discuss the implications of these findings by comparing them directly against the current state-of-the-art (SOTA) in arrhythmia detection, followed by an analysis of our own study’s limitations and directions for future work. Although our feature set is lightweight and interpretable, feature-engineered representations can be less flexible than end-to-end deep models and may miss subtle patterns learned from raw signals. *Future work* will explore hybrid designs (lightweight 1D-CNN front-end + compact classifier) and distillation to retain edge efficiency while improving representational flexibility. The clinician evaluation involved a small sample (*N* = 12) and assessed perceived clarity/trust/utility of explanations rather than real-world diagnostic impact. Participant demographics and expertise may limit generalizability (we report available characteristics; any unavailable fields are noted). Importantly, this work is not a prospective clinical trial and does not establish patient-level safety or clinical outcomes. Larger multi-center prospective studies are required before clinical deployment claims.

*Translational relevance and limitations*: While this work targets medical IoT use cases, we emphasize that our current evidence supports technical readiness and practical feasibility, not full clinical deployment. Specifically, real-world applicability is supported in two ways: (i) edge validation on an ESP32, where we quantify model size, latency, and energy consumption to demonstrate that the proposed pipeline can operate under realistic MIoT constraints (Sect. “4.5”; Table 11; Figs. 12 and 13); and (ii) a clinical utility survey of the generated interpretability reports with practicing cardiologists (Sect. “4.6.3”; Table 13; Fig. 15), indicating perceived usefulness and trust. However, this is not a prospective clinical study; therefore, future work must include prospective and multi-center validation, robustness testing across devices and acquisition conditions, calibration and failure-mode analysis under clinical workflow constraints, and post-deployment monitoring to establish safety and generalizability in routine care.

### Statistical analysis of proposed method

To strengthen statistical support using only the reported results, we provide effect-size evidence, deployment ratios, and survey uncertainty. On MIT-BIH, CLARITY-AI 2.0 shows comparable performance to the 1D-CNN benchmark (ΔAUC = + 0.003; ΔF1 = − 0.004) while showing substantial gains over the original CLARITY-AI (ΔF1 = + 0.087; ΔRecall = + 0.100). Zero-shot generalization exhibits small deltas versus the benchmark across PTB-XL and Chapman (− 0.006 and + 0.006 F1), indicating stability across datasets. Real-world feasibility is strongly supported by large on-device effect sizes on ESP32 (12.29× smaller model, 9.39× lower RAM, 11.75× faster inference). Finally, clinician feedback is reported with exact 95% binomial confidence intervals (*N* = 12), quantifying uncertainty while still indicating high agreement on trust and explanation clarity (see Tables 15, 16, 17 and 18).

**Table 15:** MIT-BIH binary anomaly detection: effect sizes vs. baselines. (Absolute Δ and relative % change from reported point estimates).

A) CLARITY-AI 2.0 vs. 1D-CNN benchmark				
Metric	1D-CNN	CLARITY-AI 2.0	Δ (Ours−Base)	% Change
Accuracy	0.985	0.989	+0.004	+0.41%
Precision	0.930	0.931	+0.001	+0.11%
Recall	0.935	0.925	−0.010	−1.07%
F1	0.932	0.928	−0.004	−0.43%
AUC	0.982	0.985	+0.003	+0.31%

B) CLARITY-AI 2.0 vs. original CLARITY-AI				
Metric	CLARITY-AI (orig)	CLARITY-AI 2.0	Δ (Ours−Base)	% Change
Accuracy	0.982	0.989	+0.007	+0.71%
Precision	0.858	0.931	+0.073	+8.51%
Recall	0.825	0.925	+0.100	+12.12%
F1	0.841	0.928	+0.087	+10.34%
AUC	0.966	0.985	+0.019	+1.97%

**Table 16:** Cross-Dataset Zero-Shot Generalization: F1 Effect Sizes. (Trained on MIT-BIH; reported F1 values).

Dataset	1D-CNN F1	CLARITY-AI 2.0 F1	Δ (Ours−Base)	% Change
MIT-BIH	0.932	0.928	−0.004	−0.43%
PTB-XL	0.891	0.885	−0.006	−0.67%
Chapman	0.855	0.861	+ 0.006	+ 0.70%

Additional AUCs reported for CLARITY-AI 2.0: PTB-XL AUC = 0.940, Chapman AUC = 0.915.

**Table 17:** ESP32 Deployment Evidence: Compression + Speedup + Memory Reduction. (Computed from reported ESP32 values).

Metric	1D-CNN	CLARITY-AI 2.0	Ratio (Base/Ours)	Reduction
Model size	4.2 MB	350 KB	12.29× smaller	91.86%
Peak RAM	1.1 MB	120 KB	9.39× lower	89.35%
Inference time	95.2 ms	8.1 ms	11.75× faster	91.49%

**Table 18:** Clinician Survey: Proportions with Exact 95% Binomial Confidence Intervals (*N* = 12). (Statistical uncertainty using only reported counts).

Survey item	Count	Proportion	95% CI (exact binomial)
SHAP report increases trust	11/12	91.7%	61.5% – 99.8%
Comfortable using it as a diagnostic aid	9/12	75.0%	42.8% – 94.5%
Anomalous explanation is clear	10/12	83.3%	51.6% – 97.9%

### IDS reproducibility details (dataset preparation, threat model, and evaluation)

To ensure reproducibility of the IDS component, we explicitly document (i) the threat model scope and assumptions, (ii) the IDS dataset preparation pipeline (sources, labeling, cleaning, and splits), and (iii) the evaluation protocol (metrics, confidence intervals, and reporting) (see Tables 19, 20 and 21).

**Table 19:** IDS Threat Model Assumptions.

Item	Included (in-scope)	Excluded (out-of-scope)
Attacker location	Remote/network-side adversary in MIoT communication	Physical access to the device
Attack types	Data manipulation (noise injection/temporal distortion), false data injection/replay, availability/battery-drain/DoS-style disruptions	Firmware/model tampering, hardware replacement
Attacker goal	Degrade inference reliability or disrupt service	Full system compromise claims
Trust integration	IDS outputs attack probability ${p}_{attackp}$; trust $T=1-{p}_{attack}$; flag as untrusted if $T<\tau$	IDS treated as proof of clinical safety

**Table 20:** IDS Dataset Preparation Pipeline (Reproducible Steps).

Step	What we do	What we save/report
1) Data sources	Use CICIoMT2024 and X-IoMTDataset	Dataset name + version/date downloaded
2) Label harmonization	Map labels to Benign vs. Attack for IDS evaluation (retain original attack types if available)	Final label schema + mapping rules
3) Cleaning	Remove invalid/empty records; enforce numeric validity; remove duplicates where applicable	Counts removed per rule
4) Splitting	Use dataset-provided splits when available; otherwise stratified train/val/test split	Split ratios + random seed
5) Leakage prevention	Ensure no overlap across splits (IDs/sessions/flows as applicable)	Verification check summary
6) Repro controls	Fix seed(s) for split and model training	Seed values + environment details

**Table 21:** IDS Evaluation Protocol (What to Compute and Report).

Category	Reported outputs
Core detection	Precision, Recall, F1-score, ROC-AUC, PR-AUC
Error trade-offs	FPR, FNR (at the reported operating threshold τ\tauτ)
Robustness under imbalance	MCC
Probability reliability	Brier score (calibration error)
Stability	ROC and PR curves with 95% confidence intervals across repeated runs
Trust decision rule	Report τ\tauτ and how it is selected (fixed, validation-tuned, or sensitivity sweep)

## Conclusion

This work presents CLARITY-AI 2.0, a lightweight, security-aware framework for Medical IoT (MIoT) cardiac monitoring. Moving away from computationally heavy deep learning, it employs a novel 40-dimensional hybrid feature representation (statistical, fiducial, wavelet, and HRV) to achieve high diagnostic accuracy on resource-constrained edge devices. The framework delivers an F1 score of 0.928 on the MIT-BIH benchmark and generalizes strongly with an AUC of 0.940 on the unseen PTB-XL dataset. Crucially, CLARITY-AI 2.0 integrates a lightweight Intrusion Detection System (IDS) to ensure data integrity against cyber-physical threats, outperforming existing baselines in robustness. For edge deployment, testing on an ESP32 microcontroller proved the system is 12× smaller, 11.7× faster, and 11.5× more energy-efficient than representative 1D-CNNs. To address the black-box nature of AI, the framework introduces a dual explainability pipeline combining SHAP attributions with a novel LLM engine to generate human-readable clinical and security reports. A user study with 12 cardiologists confirmed its practical value, with 91.7% reporting increased diagnostic trust. Ultimately, CLARITY-AI 2.0 successfully unifies efficient clinical intelligence, robust security, and clinician-centered explainability for scalable, real-world wearable healthcare.

## Data Availability

The dataset used/analyzed in this study is available on the following websites: PhysioNet 1 (Official Source): https://physionet.org/content/mitdb/1.0.0/Kaggle Mirror: https://www.kaggle.com/datasets/mondejar/mitbih-databasePhysioNet 2 (Official Source): https://physionet.org/content/ptb-xl/1.0.3/Kaggle Mirror: https://www.kaggle.com/datasets/bjoernjostein/ptbxlphysionetPhysioNet 3 (Official Source): https://physionet.org/content/ecg-arrhythmia/1.0.0/Kaggle Mirror: https://www.kaggle.com/datasets/erarayamorenzomuten/chapmanshaoxing-12lead-ecg-database CICIoMT2024 IoMT-IDS 1: https://www.unb.ca/cic/datasets/iomt-dataset-2024.html X-IoMTDataset IoMT-IDS 2: https://github.com/RuiPintoUBI/X-IoMTDataset.
